# A Genetic Basis for a Postmeiotic X Versus Y Chromosome Intragenomic Conflict in the Mouse

**DOI:** 10.1371/journal.pgen.1002900

**Published:** 2012-09-13

**Authors:** Julie Cocquet, Peter J. I. Ellis, Shantha K. Mahadevaiah, Nabeel A. Affara, Daniel Vaiman, Paul S. Burgoyne

**Affiliations:** 1 Inserm, U1016, Institut Cochin, Paris, France; 2 CNRS, UMR8104, Paris, France; 3 Université Paris Descartes, Sorbonne Paris Cité, Paris, France; 4 Department of Pathology, Mammalian Molecular Genetics Group, University of Cambridge, Cambridge, United Kingdom; 5 Division of Stem Cell Biology and Developmental Genetics, MRC National Institute for Medical Research, London, United Kingdom; University of Arizona, United States of America

## Abstract

Intragenomic conflicts arise when a genetic element favours its own transmission to the detriment of others. Conflicts over sex chromosome transmission are expected to have influenced genome structure, gene regulation, and speciation. In the mouse, the existence of an intragenomic conflict between X- and Y-linked multicopy genes has long been suggested but never demonstrated. The Y-encoded multicopy gene *Sly* has been shown to have a predominant role in the epigenetic repression of post meiotic sex chromatin (PMSC) and, as such, represses X and Y genes, among which are its X-linked homologs *Slx* and *Slxl1*. Here, we produced mice that are deficient for both *Sly* and *Slx/Slxl1* and observed that *Slx/Slxl1* has an opposite role to that of *Sly*, in that it stimulates XY gene expression in spermatids. *Slx/Slxl1* deficiency rescues the sperm differentiation defects and near sterility caused by *Sly* deficiency and vice versa. *Slx/Slxl1* deficiency also causes a sex ratio distortion towards the production of male offspring that is corrected by *Sly* deficiency. All in all, our data show that *Slx/Slxl1* and *Sly* have antagonistic effects during sperm differentiation and are involved in a postmeiotic intragenomic conflict that causes segregation distortion and male sterility. This is undoubtedly what drove the massive gene amplification on the mouse X and Y chromosomes. It may also be at the basis of cases of F1 male hybrid sterility where the balance between *Slx/Slxl1* and *Sly* copy number, and therefore expression, is disrupted. To the best of our knowledge, our work is the first demonstration of a competition occurring between X and Y related genes in mammals. It also provides a biological basis for the concept that intragenomic conflict is an important evolutionary force which impacts on gene expression, genome structure, and speciation.

## Author Summary

Both copies of a gene have normally an equal chance of being inherited; however, some genes can act “selfishly” to be transmitted to >50% of offspring: a phenomenon known as transmission distortion. Distorting genes on the X or Y chromosome leads to an excess of female/male offspring respectively. This then sets up a “genomic conflict” (arms race) between the sex chromosomes that can radically affect their gene content. Male mice that have lost part of their Y produce >50% female offspring and show over-activation of multiple genes on the X, providing strong circumstantial evidence for distortion. Here, we demonstrate the existence of a genomic conflict regulated by the genes *Slx/Slxl1* and *Sly*, present in ∼50 to 100 copies on the X and Y chromosomes respectively. SLX/SLXL1 and SLY proteins have antagonistic effects on sex chromosome expression in developing sperm and skew the offspring sex-ratio in favor of females/males. Interestingly, while deficiency of either gene alone leads to severe fertility problems, fertility is improved when both genes are deficient. We believe that the conflict in which *Slx/Slxl1* and *Sly* are involved led to the amplification of X and Y genes and may have played an important role in mouse speciation.

## Introduction

Transmission distorters (TDs), also known as segregation distorters or meiotic drivers, are genetic elements that are transmitted to the next generation with a higher frequency than the expected 1∶1 Mendelian inheritance ratio. TDs have the tendency to accumulate in low recombination regions where tight linkage allows cooperation between TDs and responder genes to evolve, as seen in the mouse *t*-complex [1] [for recent reviews see [2], [3]]. The non-recombining region of the heteromorphic sex chromosomes is the largest genomic example of recombination suppression [4], with the consequent potential for TDs to arise and distort the population sex ratio. Theory predicts that an unlinked suppressor of the sex ratio distortion (whether autosomal or on the other sex chromosome) would rapidly be selected for to restore the Fisherian 1∶1 sex ratio [5]. A subsequent evolutionary arms race between the distorter and its suppressor may follow and lead to repeated bouts of amplification of the genes involved in this intragenomic conflict [6]. In *Drosophila*, the X- and Y-encoded multicopy genes *Stellate* and *Suppressor of Stellate* are believed to illustrate the genomic conflict theory since deletions of *Su(Ste)* locus lead to a derepression of *Stellate* associated with a distorted sex ratio towards an excess of females; but to date it remains unclear whether or not *Stellate* is a transmission distorter [7], [8]. Intragenomic conflicts over sex chromosome transmission are predicted to have influenced genome structure, gene expression and speciation [2], [3]. Several cases of sex chromosome transmission distortion have been reported in the literature but they mostly concern Drosophila species [2], [9]–[14] and remain poorly characterized in mammals. Sex ratio segregation distortion may be more frequent than observed as the distortion is often masked by the presence of a suppressor in wild-type (WT) populations [2], [9]–[15].

In the mouse, the existence of an intragenomic conflict between X- and Y-linked genes has long been suggested: males with a partial deletion of the male specific region of the Y long arm (MSYq) produce offspring with a sex ratio skewed towards females [16], suggesting that MSYq encodes a factor(s) suppressing sex ratio distortion. MSYq consists of multicopy gene families, present in ∼60 to 100 copies [17]–[24], many of which possess X-linked multicopy homologous genes [20], [25], [26]. This has been considered a manifestation of a conflict between an X-encoded TD and a Y-encoded suppressor that remain to be identified [16], [20], [26], [27].

We have previously shown that the MSYq-encoded multicopy gene *Sly* (*Sycp3-like Y-linked*) represses the postmeiotic expression of X and Y genes [19]. *Sly*-deficient males – also known as shSLY males since they carry a short hairpin RNA-expressing transgene which triggers the specific degradation of *Sly* transcripts by RNA interference – present a remarkable up-regulation of sex chromosome genes in postmeiotic germ cells (spermatids) associated with a loss of repressive epigenetic marks, such as trimethylated histone H3 (H3K9me3) and CBX1 [19]. SLY therefore limits sex chromosome expression *via* the recruitment/maintenance of repressive epigenetic marks to post meiotic sex chromatin (PMSC) and has been proposed to associate with the sex chromosomes through its Cor1 domain – a domain thought to mediate chromatin interactions (Conserved Domain Database from the National Center for Biotechnology Information, http://www.ncbi.nlm.nih.gov/Structure/cdd/cddsrv.cgi?uid=147120).

Interestingly, *Slx* and *Slxl1*, two multicopy X-linked genes related to *Sly* [25] have been co-amplified with *Sly* during the evolution of the mouse genome [18], [20] and are among the genes that are up-regulated when *Sly* expression is reduced/absent [19]. Using a strategy of transgenically-delivered short hairpin RNA similar to the one previously used to disrupt the function of *Sly*, we have recently produced *Slx/Slxl1*-deficient mice (also known as shSLX mice). This study has shown that *Slx/Slxl1* are indispensable for normal sperm differentiation, and that *Slx/Slxl1* deficiency leads to the deregulation of a number of autosomal genes [28]. Moreover, both SLY and SLXL1 proteins have now been shown to interact with the acrosomal protein DKKL1 [21], [29].

In the present study we show that SLX/SLXL1 and SLY proteins have antagonistic effects on gene expression for both the sex chromosomal genes deregulated in shSLY and the set of autosomal genes deregulated in shSLX, and furthermore have antagonistic effects on offspring sex ratio. Our data demonstrate that *Slx/Slxl1* and *Sly* are involved in a postmeiotic intragenomic conflict; we propose this phenomenon has had a strong impact on the structure and epigenetic regulation of the sex chromosomes, and may also have influenced the evolution of hybrid sterility in the mouse lineage.

## Results

### In *Sly*-deficient mice, SLX/SLXL1 proteins relocate to the nuclear sites vacated by SLY proteins

**Figure 1:**
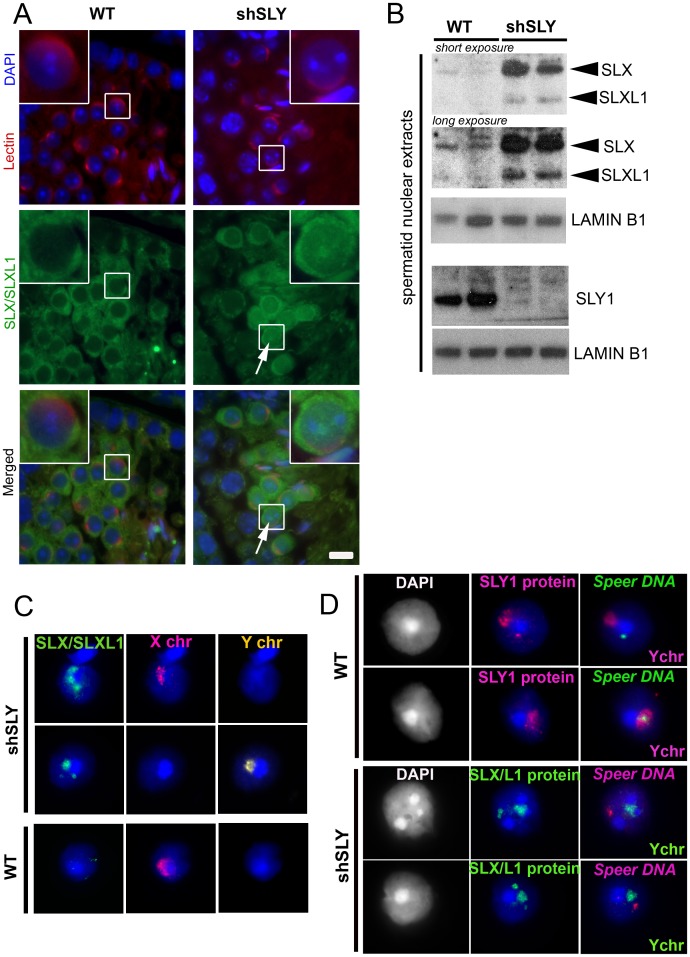
SLX/SLXL1 proteins behave similarly to SLY in its absence. A) Immunofluorescence detection of SLX/SLXL1 protein (green) in wild-type (WT) and *Sly*-deficient (shSLY) testicular sections. DAPI (blue) was used to stain nuclei and lectin-PNA (red) was used to stain acrosomes in order to determine tubular stage. The inset represents a 3× magnification. Pictures were taken using the same image capture parameters. Scale bar indicates 10 µm. B) Western blot detection of SLX/SLXL1 proteins in nuclear extracts from shSLY and WT round spermatids. SLY1 antibody was used on the same extracts to confirm the absence of SLY protein in the shSLY nuclear fraction. *Sly* gene encodes two alternative splice variants (*Sly1* and *Sly2*) which are predicted to be translated into a long and a short protein isoform (SLY1 and SLY2), but only SLY1 proteins have been detected so far and it remains unclear whether *Sly2* transcripts are translated [21]. LAMIN-B1 detection was used as a loading control. C) Immunofluorescence detection of SLX/SLXL1 protein (green) in shSLY and WT round spermatid nuclei. DAPI (blue) was used to stain nuclei. X and Y chromosome painting were performed sequentially. A strong SLX/SLXL1 signal was observed in the majority of shSLY spermatid nuclei (76%). This signal colocalized with either sex chromosome in 96.5% of the cases. No signal could be detected in the majority of WT round spermatid nuclei (84%). D) Immunofluorescence detection of SLY1 (pink) or SLX/SLXL1 (green) protein in WT or shSLY round spermatids. Hybridization with a DNA probe detecting *Speer* gene cluster was subsequently performed, followed by Y chromosome painting. DAPI (white or blue) was used to stain nuclei. SLY1 protein colocalized with *Speer* gene cluster in 78.5% of WT spermatids while SLX/SLXL1 proteins colocalized with *Speer* gene cluster in 73% of shSLY spermatids.

In normal males, SLX/SLXL1 proteins are located in the cytoplasmic compartment of spermatids [28], whereas SLY is additionally detected in the spermatid nucleus where it has been shown to colocalize with the X and Y chromosomes [19]. When performing immunofluorescence detection of SLX/SLXL1 proteins on spermatids devoid of SLY protein (i.e. on shSLY testicular sections), we observed an augmented SLX/SLXL1 signal in the cytoplasm compared to controls (WT) – confirming up-regulation at the protein level – and some signal in shSLY round spermatid nuclei that was not visible in WT (Figure 1A). The presence of SLX/SLXL1 proteins in shSLY spermatid nuclei was confirmed by Western blot analyses of nuclear fractions (Figure 1B). We then investigated in more detail the nuclear localization of SLX/SLXL1 in the context of *Sly* deficiency. The vast majority of shSLY spermatid nuclei showed a strong SLX/SLXL1 signal (280/369, 76%) (Figure 1C and Figure S1). This signal colocalized with the postmeiotic sex chromatin (PMSC, i.e. the X or the Y chromosome since spermatids are haploid) in 96.5% of round spermatids (82/85; 32/34 for X-bearing and 50/51 for Y-bearing spermatids). In comparison, 84% of WT round spermatid nuclei (265/316) did not have any SLX/SLXL1 signal. The nuclear SLX/SLXL1 signal observed in the remaining ∼16% of WT round spermatid was very weak when compared to the nuclear signal in shSLY round spermatids but appeared to colocalize with the PMSC in the majority of the cases (Figure S1).

In addition to colocalizing with the PMSC, foci of SLX/SLXL1 proteins were observed outside the sex chromatin, reminiscent of the SLY signal present in the nucleus of WT spermatids [19]. We have since established that these ‘ectopic’ SLY sites include a ∼14 Mb cluster of 7 *Speer* genes on chromosome 5 that are up-regulated in shSLY spermatids. As a result, SLY immunofluorescence followed by fluorescent hybridization of a *Speer* DNA probe (DNA FISH) showed that, in the majority of WT round spermatids (107/136, 78.5%), SLY protein colocalized with the *Speer* DNA FISH signal (Figure 1D). We next looked at SLX/SLXL1 proteins in *Sly*-deficient round spermatids and observed that they colocalized with the *Speer* gene cluster in 73% of the cases (130/178) (Figure 1D). Thus, in the absence of SLY, SLX/SLXL1 proteins colocalize with the sex chromatin and with the autosomal *Speer* gene cluster, mimicking the pattern observed for SLY protein in WT spermatids.

### Transgenic delivery of shSLX and shSLY short hairpin RNAs leads to a dramatic reduction in *Slx/Slxl1* and *Sly* RNA and protein levels

**Figure 2:**
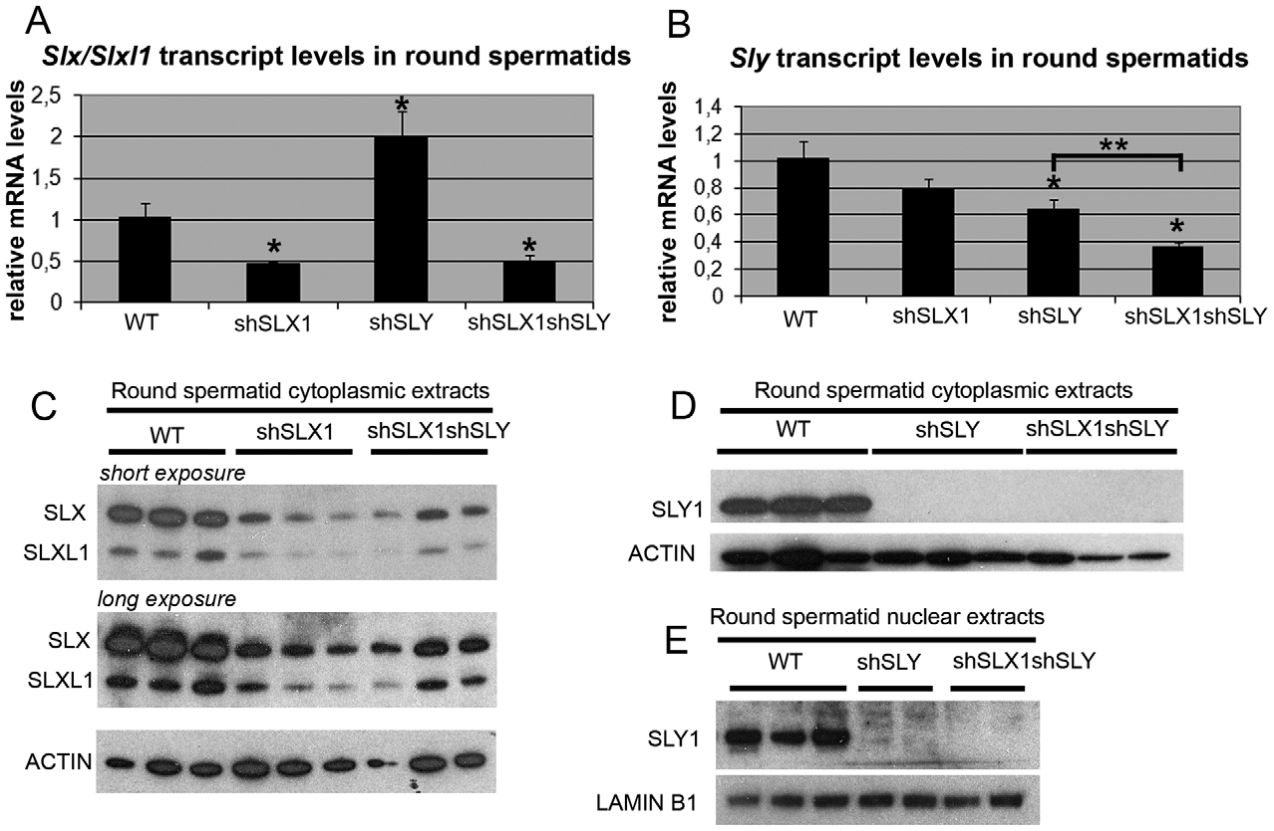
The combination of shSLX and shSLY transgenes produces an efficient knockdown of *Slx/Slxl1* and *Sly* genes. A–B) Real time PCR quantification of *Slx*/*Slxl1* (*Slx-all* primers) (A) and *Sly* (*Sly1* and *Sly2* variants) (B) transcript levels in WT, shSLX1, shSLY and shSLX1shSLY round spermatids. The y-axis indicates the level of expression compared to WT after normalization with *Acrv1* (2^ΔΔCt^ ± standard errors). The reduction in *Slx/Slxl1* transcript level was similar in shSLX1shSLY males and in shSLX1 siblings. As observed before [19], *Slx/Slxl1* transcript level was found increased in shSLY males. One asterisk indicates significant difference from WT (p<0.05; t test on ΔΔCt values). *Sly* knockdown was even stronger in shSLX1shSLY males compared to shSLY siblings [two asterisks indicate significant difference between shSLX1shSLY and shSLY (p = 0.02; t test on ΔΔCt values)]. C–E) Western blot detection of SLY1, SLX and SLXL1 proteins in nuclear and cytoplasmic fractions from WT, shSLY, shSLX1 and shSLX1shSLY round spermatids. LAMIN B1 and ACTIN detection were used as loading controls for nuclear and cytoplasmic fractions, respectively. No SLY1 protein could be detected in shSLY or in shSLX1shSLY samples.

We then wondered if the localization of SLX/SLXL1 proteins to the PMSC in the absence of SLY also affects postmeiotic sex chromosome gene expression. To address this question, we generated males that were deficient for SLX/SLXL1 and SLY proteins: we produced males carrying shSLY (*Sly* specific short hairpin RNA) transgene [19] together with one or two shSLX (*Slx/Slxl1* specific short hairpin RNA) transgenes, shSLX1 and/or shSLX2 [28]. Firstly, we checked the efficiency of *Slx/Slxl1* and *Sly* knockdowns in round spermatids from males carrying shSLX1 and shSLY transgenes (hereafter named shSLX1shSLY males). The reduction in *Slx/Slxl1* transcript level was similar in shSLX1shSLY males and in shSLX1 siblings, while *Sly* knockdown was even stronger in shSLX1shSLY males compared to shSLY siblings (Figure 2A and 2B). *Sly* transcript quantification included both alternative splice variants (*Sly1* and *Sly2*) [21] which were knocked-down with the same efficiency [19]. No SLY1 protein could be detected in shSLY or in shSLX1shSLY tissues (Figure 2D–2E). To date it remains unclear whether *Sly2* transcripts are translated since anti-SLY1 antibody cannot detect SLY2 protein [21]. The discrepancy between transcript and protein levels is likely due to the presence of non-coding *Sly* transcripts, as previously observed [19]. Reduction in SLX and SLXL1 proteins was similar in shSLX1shSLY males and in shSLX1 siblings (Figure 2C). We also produced shSLX1/2shSLY males that carry the two shSLX transgenes along with the shSLY transgene. As expected, shSLX1/2shSLY males showed a very efficient knockdown of *Slx* and *Slxl1* (Figure S2A); *Sly* knockdown in these males was similar to that in shSLX1shSLY males (Figure S2B). Thus, the combination of shSLX and shSLY transgenes gives an efficient knockdown of *Slx/Slxl1* and *Sly* genes; the resulting transgenic males are therefore deficient for *Slx/Slxl1* and *Sly* transcripts and proteins (hereafter named *Slx/y*-deficient males).

### In *Sly*-deficient spermatids, SLX/SLXL1 proteins increase sex chromosome gene expression associated with a reduction of H3K9me3 marks on PMSC

**Figure 3:**
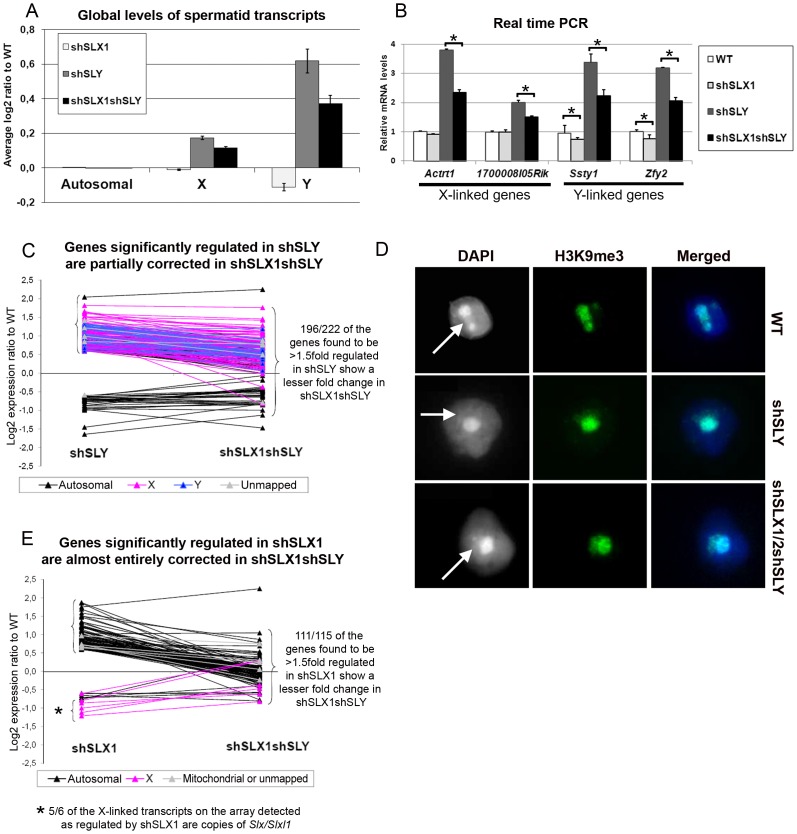
SLX/SLXL1 and SLY have opposite effects on gene expression and on the recruitment/maintenance of H3K9me3 on the sex chromatin. A) Representation of the microarray results obtained for *Slx/Slxl1*-deficient (shSLX1), *Sly*-deficient (shSLY) and *Slx/y*-deficient (shSLX1shSLY) compared to WT spermatids. B) Real time PCR quantification showed that transcript levels of X-encoded (*Actrt1* and *1700008I05Rik*) and of Y-encoded genes (*Ssty1* and *Zfy2*) were lower in shSLX1shSLY than in shSLY spermatids. Transcript levels were also lower in shSLX1 spermatids compared to WT spermatids. The y-axis indicates the level of expression compared to WT after normalization with *Acrv1* (2^ΔΔCt^ ± standard errors). One asterisk indicates significant difference from corresponding shSLY or WT value (t test on ΔΔCt values; p<0.05). C) Graphic representation of the expression ratio relative to WT for the 222 genes showing greater than 1.5 fold-change in shSLY. Most genes affected by shSLY are sex-linked and up-regulated [19]. The majority of them (196/222) were corrected to some degree by addition of the shSLX transgene (in shSLX1shSLY). For 46 of these genes, the difference between shSLY and shSLX1shSLY was itself statistically significant. D) Immunofluorescence detection of H3K9me3 (green) in WT, shSLY and shSLX1/2shSLY round spermatid nuclei. DAPI (white or blue) was used to stain nuclei. The round DAPI-dense structure is the chromocenter. The less DAPI-dense structure at the periphery of the chromocenter is the postmeiotic sex chromatin (PMSC) and is indicated by an arrow. Pictures were taken using the same image capture parameters. Note the decreased H3K9me3 signal on the sex chromatin of *Sly*-deficient spermatids; this is almost completely restored by *Slx/Slxl1* deficiency (in shSLX1/2shSLY spermatids). See also Figure S4. E) Graphic representation of the expression ratio relative to WT for the 115 genes showing greater than 1.5 fold-change in shSLX1. Most genes affected by shSLX1 are autosomal and up-regulated [28]. Note that almost all of them (111/115) have a lower fold change in shSLX1shSLY than in shSLX1. For 91 of these genes, the difference between shSLX1 and shSLX1shSLY was itself statistically significant.

We then performed microarray transcriptome analyses on *Slx/y*-deficient purified round spermatids and compared these results to those obtained from *Sly*-deficient and from WT round spermatids (Figure 3 and Figure S3). The up-regulation of X- and Y-encoded spermatid transcripts was significantly less pronounced in *Slx/y*-deficient males than in *Sly*-deficient males (Figure 3A–3C). Specifically, 222 genes showed a greater than 1.5 fold-increase in *Sly*-deficient spermatids relative to WT, and 196 of them were corrected to some degree by the additional *Slx/Slxl1* deficiency (i.e. in *Slx/y*-deficient spermatids). As a Y-encoded gene, *Sly* itself is affected by *Slx/Slxl1* knockdown and thus is expressed at a lower level in *Slx/y*-deficient males than in *Sly*-deficient males (Figure 2B and Figure S3). The microarray findings were confirmed for several representative X and Y genes by real time PCR (Figure 3B and Figure S2C). These opposite effects of *Sly* and *Slx/Slxl1* deficiency show that, in the absence of SLY protein, SLX/SLXL1 proteins localize to PMSC where they increase sex chromosome gene expression; when both SLX/SLXL1 and SLY proteins are reduced/absent in PMSC (in *Slx/y*-deficient males), the level of X- and Y- encoded transcripts is closer to the WT value. It is worth noting that while *Slx/Slxl1* deficiency significantly reduces the up-regulation of XY genes induced by *Sly* deficiency, it does not bring expression all the way back down to WT levels. This may indicate that *Slx/Slxl1* knockdown is not sufficient to fully compensate for the effect of *Sly* deficiency; alternatively it may be that in the WT MF1 laboratory strain, the combined effect of the presence of both SLX/SLXL1 and SLY is a net reduction of XY expression level, thus leading to a net increase when both genes are deficient.

The up-regulation of X- and Y-encoded spermatid genes in *Sly*-deficient spermatids has been shown to be concurrent with a diminution of the repressive epigenetic marks (such as H3K9me3) normally associated with PMSC [19]. We therefore decided to study these repressive marks in *Slx/y*-deficient spermatids, and observed that H3K9me3 staining on PMSC (as compared to H3K9me3 chromocenter staining) was significantly higher (p = 0.00003) in *Slx/y*-deficient spermatids than in *Sly*-deficient spermatids (average staining intensity: 0.59 and 0.51 respectively), and closer to but significantly different from the WT value (average staining intensity in WT: 0.65, p = 0.003) (Figure 3D and Figure S4 for quantification). Therefore *Slx/Slxl1* deficiency partially compensates the loss of H3K9me3 marks induced by *Sly* deficiency. These results correlate with the global effect of *Slx/Slxl1* transcript knockdown on sex chromosome expression and suggest that SLX/SLXL1 and SLY proteins compete in spermatids for access to PMSC where they have activator and repressive effects respectively, at the whole-chromosome level.

We then compared the transcriptomes of WT, *Slx/Slxl1*-deficient and *Slx/y*-deficient spermatids. This revealed a 10% reduction in Y transcription in *Slx/Slxl1*-deficient spermatids compared to WT that was not seen in an earlier study [28] (Figure 3A–3B). This reduction is congruent with our observation of some SLX/SLXL1 proteins in a small number of WT spermatid nuclei (Figure 1B and Figure S1); this small fraction of SLX/SLXL1 proteins most likely increases sex chromosome gene expression in the nucleus of WT spermatids, while the loss of these proteins leads to a slight reduction of XY expression in *Slx/Slxl1*-deficient spermatids. A faint reduction of expression was observed for some X genes (for instance *Actrt1*, see Figure 3B) but this did not significantly differ from the WT value.

### *Sly* knockdown corrects the gene deregulation induced by *Slx/Slxl1* deficiency

We have previously shown that *Slx/Slxl1* deficiency leads to delay in spermatid elongation and sperm release, associated with the deregulation (principally the up-regulation) of 115 genes, the majority of which are located on the autosomes. Given that SLX/SLXL1 proteins are almost entirely cytoplasmic in wild type, we proposed that these transcriptional changes were a manifestation of “cytoplasmic” defects, rather than a direct effect of SLX/SLXL1 proteins on autosomal gene expression; for instance, an as yet unidentified cytoplasmic partner of SLX/SLXL1 could mediate the transcriptional changes that are necessary for normal spermatid elongation, or it may be that the transcriptional changes seen reflect an altered cellular proportion of different step spermatids in shSLX [28]. In the present study, we compared microarray results from *Slx/Slxl1*-deficient and *Slx/y*-deficient spermatids and, surprisingly, observed that most of the genes deregulated by *Slx/Slxl1* deficiency were less affected in *Slx/y*-deficient spermatids (111/115 genes, Figure 3E and Figure S5). Therefore, *Sly* knockdown corrects the deregulation of autosomal genes induced by *Slx/Slxl1* (with autosomal gene expression values close to WT levels in *Slx/y*-deficient spermatids; Figure 3E and Figure S5). These results show that SLX/Y proteins have opposite regulatory effects on autosomal gene expression as well as on sex chromosome gene expression.

### *Slx/y*-deficient males have better reproductive parameters and overall fertility than males that are deficient for either *Slx/Slxl1* or for *Sly*

**Figure 4:**
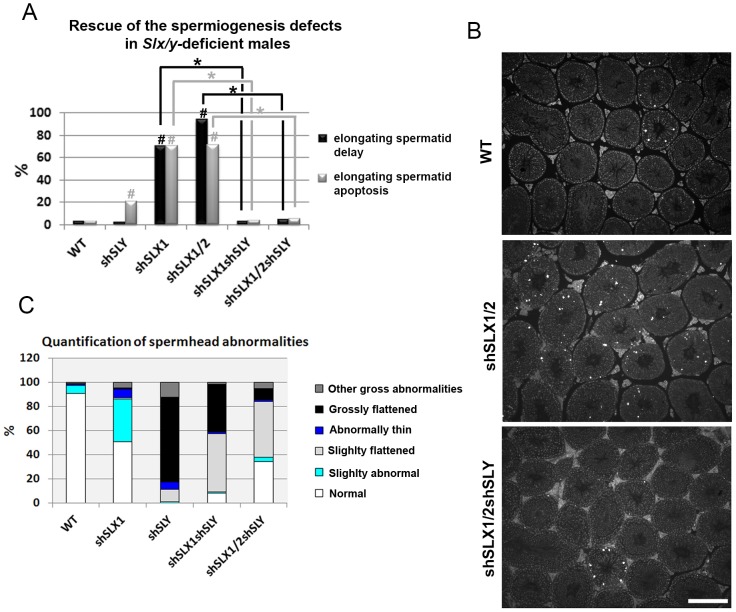
*Slx/y*-deficient males have fewer spermiogenesis defects than males that are deficient for either *Sly* or *Slx/Slxl1* alone. A) Bar graph representing the percentage of tubules containing apoptotic elongating spermatids measured by TUNEL assay (in grey) and the percentage of tubules containing delayed elongating spermatids (in black). Hatch symbol (#) indicates significant difference from WT (ANOVA, p<0.0001). One asterisk indicates significant difference between values obtained for *Slx/y*-deficient males (shSLX1shSLY or shSLX1/2shSLY) and *Slx/Slxl1*-deficient siblings (shSLX1 or shSLX1/2) (ANOVA, p<0.00001). B) Representative black and white TUNEL pictures of WT, shSLX1/2 and shSLX1/2shSLY testicular sections. Note the presence of apoptotic cells (TUNEL+ cells in white) in most shSLX1/2 seminiferous tubules, while in WT and in shSLX1/2shSLY they are largely restricted to stage XII tubules (and correspond to metaphasic cells undergoing normal apoptosis). Scale bar represents 200 µm. C) Bar graph representing the percentage of sperm head abnormalities in *Slx/Slxl1*-deficient, *Sly*-deficient and *Slx/y*-deficient males (see also Figure S6). The percentage of total abnormal sperm heads is significantly lower in *Slx/y*-deficient males (shSLX1shSLY and shSLX1/2shSLY) than in *Sly*-deficient males (shSLY). In particular, the number of grossly flattened spermheads – which are specifically observed in *Sly*-deficient males – is reduced (ANOVA, p<0.001). ShSLX1/2shSLY males also show a significant decrease in this percentage compared to shSLX1shSLY males (ANOVA, p<0.03). The number of slightly abnormal sperm heads – which are specifically observed in *Slx/Slxl1*-deficient males – is also reduced in *Slx/y*-deficient males compared to *Slx/Slxl1*-deficient males (ANOVA, p<0.0008). For this category of sperm abnormalities, *Slx/y*-deficient males did not significantly differ from WT.

Our microarray results demonstrate that the deregulation of sex chromosome-linked or autosomal genes observed in *Sly*-deficient or in *Slx/Slxl1*-deficient spermatids respectively, is corrected in *Slx/y*-deficient spermatids; we therefore compared the reproductive parameters of *Slx/y*-deficient males with those from males that are singly deficient for either *Slx/Slxl1* or for *Sly*. Firstly, *Slx/y*-deficient males had significantly improved sperm numbers (Table 1). This was particularly striking for the comparison between shSLX1/2 and shSLX1/2shSLY males: shSLX1/2 males had dramatically reduced spermatozoa numbers but the addition of shSLY transgene to this genotype increased the number of sperm produced ∼50-fold (Table 1). Low sperm count in shSLX males was attributed to the apoptosis of delayed elongating spermatids [28]. We therefore analyzed spermatid elongation delay and apoptosis in *Slx/y*-deficient males and in their *Slx/Slxl1*-deficient siblings. Remarkably, while *Slx/Slxl1*-deficient males presented a high number of delayed and apoptotic elongating spermatids, *Slx/y*-deficient models did not significantly differ from WT (Figure 4A–4B). The spermatozoa morphology of *Slx/y*-deficient males was also much improved compared to that of *Slx/Slxl1*- or *Sly*-deficient males (Figure 4C and Figure S6). Finally, we compared the fertility of *Slx/y*-deficient males with *Slx/Slxl1*- or *Sly*-deficient siblings: *Slx/y*-deficient males had overall better fertility than males that are deficient for either *Slx/Slxl1* or *Sly*, with reproductive parameters close to WT values (Table 1). Strikingly, the addition of *Sly* deficiency was able to reverse the sterility observed in *Slx/Slxl1* deficient-males (line shSLX1/2) (Table 1). All in all, males that were deficient for both *Slx/Slxl1* and *Sly* had considerably better reproductive parameters than males that were deficient for *Slx/Slxl1* or *Sly* alone.

**Table 1:** Analysis of the reproductive parameters of *Slx/y*-deficient mice compared to controls.

	WT	shSLY	shSLX1	shSLX1/2	shSLX1shSLY	shSLX1/2shSLY
Average number of litters per male ± standard errors	5.8±0.4	1.9*±0.7	1.9*±0.7	0*	3.1^c^±1.1	4.8^a^^,^ b ^,^ c±0.6
Average number of offspring per male ± standard errors	48.4±3.8	8.8*±4.7	10.4*±4.4	0*	18.4*±9.3	27.3* c±6.6
Average litter size ± standard errors	9.1±0.7	2.2*±1.1	2.4*±1.8	0*	3.6* c±1.2	5.8* c±1.2
Sperm count/cauda (mean values x10^−6^ ± standard errors)	14.4±1.6	5.5*±1.1	2.1*±0.6	0.2*±0.1	11.7^a^^,^ b ^,^ c±0.9	8.8* a ^,^ b ^,^ c±0.5

* significant difference from WT (t-test,p<0.02);

a significant difference from shSLY (t-test,p<0.03);

b significant difference from shSLX1 (t-test,p<0.02);

c significant difference from shSLX1/2 (t-test,p<0.03).

These analyses show that *Sly* deficiency almost completely rescues the defects and gene deregulation induced by *Slx/Slxl1* deficiency, while *Slx/Slxl1* knockdown only partially rescues those subsequent to *Sly* deficiency. This may be due to a different knockdown efficiency: indeed, no SLY1 protein can be detected in *Slx/y*-deficient samples while some SLX/SLXL1 proteins remain (Figure 2C–2E).

### *Slx/Slxl1* deficiency causes a sex ratio distortion in favor of males that is restored by *Sly* deficiency

We previously reported a tendency of an excess of females in the progeny of *Sly*-deficient males (7.7% excess of females, Chi-square p = 0.0569) [19]. While analyzing the fertility of our transgenic lines, we observed that *Slx/Slxl1*-deficient males (i.e. shSLX1) yielded an offspring sex ratio of 40% (74/187) female progeny, compared to a ratio of 51% (234/461) in WT siblings. This represents a statistically significant sex ratio distortion of 11% in favour of male offspring (Chi-square p = 0.006). Importantly, a normal sex ratio was restored by the addition of *Sly* deficiency: *Slx/y*-deficient males produced an offspring sex ratio of 50% (103/208) female progeny that did not differ from WT and was also significantly different from the offspring sex ratio of shSLX1 males (Chi-square p = 0.03). These data show that both *Slx/Slxl1* and *Sly* affect the transmission of X- and Y-bearing gametes, *Slx/Slxl1* favouring X transmission while *Sly* favours Y transmission.

## Discussion

### SLX/SLXL1 and SLY proteins have antagonistic effects on the expression of two distinct sets of genes

In recent years, we have identified the transcriptional consequences of *Sly* and *Slx/Slxl1* deficiency, and related these to the observed phenotypes in terms of spermatid development, sperm morphology and offspring sex ratio [19], [28]. Remarkably, we now show that in dual shRNA knockdown models where both genes are deficient, the transcriptional and phenotypic consequences of the individual knockdown are dramatically ameliorated, correcting the X/Y/*Speer* up-regulation and sperm shape abnormalities seen in *Sly*-deficient spermatids; the autosomal gene up-regulation, spermatid elongation delay and apoptosis, and sperm shape abnormalities seen in *Slx/Slxl1*-deficient spermatids; and improving fertility in both cases.

Strikingly, however, two different and almost entirely non-overlapping sets of genes are affected by the mutual antagonism of SLX/SLXL1 and SLY. In this discussion, we refer to “Group 1” genes as the set of X/Y/*Speer* genes up-regulated in *Sly*-deficient spermatids and (partially) corrected in the dual knockdown, and “Group 2” genes as the set of metabolism-related autosomal genes up-regulated in *Slx/Slxl1*-deficient spermatids and (almost fully) corrected in the dual knockdown.

### Group 1 genes: Nuclear consequences of antagonism between SLX/SLXL1 and SLY

*Sly* regulates the epigenetic repression of post meiotic sex chromatin (PMSC) and a few specific autosomal genes such as the *Speer* cluster. In the nucleus, SLY appears to act *via* the recruitment/maintenance of the repressive heterochromatin marks CBX1 and H3K9me3, which consequently limits the expression of X and Y genes in spermatids, among which are its X-linked homologs *Slx* and *Slxl1* [19]. Here, we show that, in the absence of SLY, SLX/SLXL1 proteins relocate to the nuclear sites (both sex-linked and autosomal) vacated by SLY proteins. It is unlikely that SLX/SLXL1 nuclear localization in *Sly*-deficient spermatids is solely a consequence of increased SLX/SLXL1 protein abundance, since there is no clear enrichment in nuclear SLX/SLXL1 proteins in spermatids of transgenic mice overexpressing SLX or SLXL1 (our unpublished preliminary data).

Moreover, in the double transgenic model (*Slx/y*-deficient males) where SLX/SLXL1 family members are also reduced/absent, XY gene expression, *Speer* expression and the intensity of H3K9me3 marks on the sex chromatin are closer to normal values. This indicates that SLX and/or SLXL1 have consequences both for transcriptional activity and for histone modification when present on sex chromatin, and that these are directly opposed to the effects of SLY. We therefore propose that SLX/SLXL1 and SLY proteins compete for access to nuclear sites in spermatids, where they act as positive and negative transcriptional regulators respectively. We cannot at this point say precisely where the competition occurs: it may be directly at the level of chromatin binding within the nucleus, or SLX/SLXL1 and SLY may compete for access to factors affecting nuclear import. We note that SLX and SLXL1 proteins lack nuclear localization signals (NLS) while SLY NLS is mutated/truncated [22], [25]; as such they probably depend on other interacting factors to enter the nucleus. It also remains possible that the competition is mediated indirectly: for example, SLY could affect SLX/SLXL1 intracellular localization *via* regulating the expression of a third factor controlling SLX/SLXL1 access to the nuclear sites.

### Group 2 genes: Cytoplasmic consequences of antagonism between SLX/SLXL1 and SLY

*Slx/Slxl1* deficiency has been shown to increase the level of ∼100 autosomal transcripts which code for proteins of the cytoskeleton and the extracellular matrix, or are implicated in various cytoplasmic processes (i.e. energy production, lipid metabolism, ubiquitin-mediated degradation, etc.) [28]. These transcriptional effects are corrected in *Slx/y*-deficient males, suggesting that these changes may also be manifestations of the same nuclear/chromatin regulatory antagonism exhibited by Group 1 genes, perhaps *via* relocation of repressive factors from sex chromatin to autosomal locations and vice versa. There are, however, three significant objections to this interpretation. Firstly, as noted previously, in WT spermatids SLX/SLXL1 are predominantly cytoplasmic proteins, and the levels in the nucleus are almost undetectable: it is hard therefore to see i) how *Slx/Slxl1* knockdown could directly induce widespread transcriptional changes, ii) what would then be the function of the abundant SLX/SLXL1 proteins in the cytoplasm. Secondly, this interpretation would require not only that SLX/SLXL1 act simultaneously as transcriptional activators of Group 1 genes and as transcriptional repressors of Group 2 genes, but that SLY has the reverse effect in both cases: it is challenging to imagine a mechanism that could explain this. Thirdly, if both Group 1 and Group 2 gene effects are a manifestation of the changing balance of SLX/Y proteins in the nucleus and/or of a relocation of repressive factors from the sex chromosomes to autosomes, then both groups of genes would be expected to change together. This is not the case: Group 1 genes are affected in shSLY but not in shSLX, and Group 2 genes vice versa.

**Figure 5:**
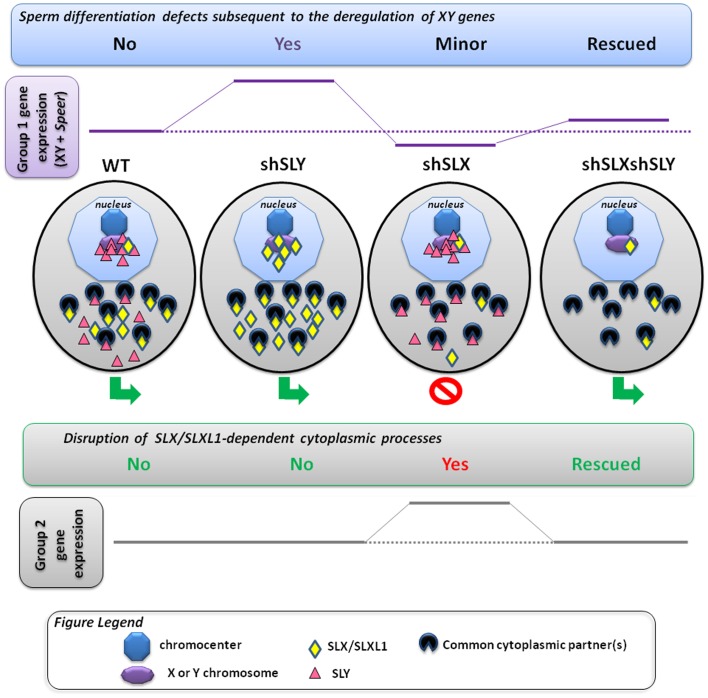
Model presenting how SLX/SLXL1 and SLY proteins have antagonistic effects in the spermatid nucleus and cytoplasm. In the spermatids, SLX/SLXL1 (yellow lozenges) and SLY (pink triangles) proteins have antagonistic effects i) in the nucleus, on the expression of XY genes and of a few autosomal genes, such as *Speer* (group 1 genes); ii) in the cytoplasm, on the regulation of metabolic processes which secondarily causes a deregulation of ∼100 autosomal genes (group 2 genes). i) In WT, SLY proteins are located in both the nucleus and cytoplasm, while SLX/SLXL1 proteins are almost exclusively in the cytoplasm. The nuclear fraction of SLY proteins colocalizes with the sex chromosomes and the autosomal *Speer* gene cluster, and represses their expression. A very small fraction of SLX/SLXL1 proteins also appears to colocalize with the sex chromatin. In *Sly*-deficient spermatids (shSLY), SLX/SLXL1 proteins relocate to the nuclear sites (both sex-linked and autosomal) vacated by SLY proteins; however, SLX/SLXL1 proteins have an opposite effect to that of SLY, and activate XY gene expression. This is associated with a reduction in the repressive epigenetic mark H3K9me3 on the sex chromatin (purple octagon), and produces sperm differentiation defects such as spermhead abnormalities, shedding delay, motility defects and subsequent male infertility. In *Slx/Slxl1*-deficient spermatids (shSLX), the absence of SLX/SLXL1 nuclear proteins has only minor effect on gene regulation, since it does not change SLY localization profile. There is only a slight reduction in XY transcription, congruent with the idea that SLX/SLXL1 is a transcription activator sharing the targets of SLY when present in the nucleus. In the double knock-down (shSLXshSLY), *Slx/Slxl1* deficiency almost fully abrogates the effects of *Sly* knockdown: in shSLXshSLY spermatids, group 1 gene expression and repressive epigenetic marks are close to WT values. This is correlated with a rescue of SLY-dependent sperm differentiation defects. In sum, in the nucleus, the experimental observations indicate that SLX/SLXL1 competes with SLY at the level of sex chromatin regulation: SLY acts as a repressor while SLX/SLXL1 acts as a positive regulator. ii) *Slx/Slxl1* deficiency induces various spermiogenic defects (such as spermatid elongation delay and apoptosis, reduced sperm count, abnormal head to tail connections of the spermatozoa and subsequent male infertility) associated with an up-regulation of ∼100 autosomal genes which code for proteins of the cytoskeleton, the extracellular matrix, or implicated in various metabolic processes (i.e. group 2 genes). Since SLX/SLXL1 proteins are predominantly cytoplasmic in WT spermatids, we propose that this gene deregulation is a manifestation of the spermiogenesis defects occasioned by *Slx/Slxl1* deficiency, and not a direct effect of SLX/SLXL1 proteins on autosomal gene transcription. In the case of *Sly* deficiency, group 2 gene expression is unaffected; however, in the double knock-down, *Sly* deficiency corrects SLX/SLXL1-dependent phenotypes which abrogates the subsequent group 2 gene up-regulation. This means that SLY protein has a cytoplasmic role, opposing that of SLX/SLXL1. This antagonism could be mediated *via* interaction with (a) common partner(s) in the cytoplasm; the absence of competition between SLX/SLXL1 and SLY proteins in the dual knockdown model would explain the absence of defects. In sum, SLX/SLXL1 and SLY proteins apparently compete in the cytoplasm for the regulation of spermiogenic processes. The functional role of SLX/SLXL1 could be to prevent the access of SLY to cytoplasmic proteins that are necessary for spermiogenesis.

For this reason, we favour our existing interpretation that Group 2 gene deregulation is a manifestation of the spermiogenesis defects occasioned by cytoplasmic *Slx/Slxl1* deficiency (i.e. spermatid elongation delay and apoptosis, reduced sperm count, abnormal head to tail connections of the spermatozoa and male infertility) [28], and is not a direct effect of SLX/SLXL1 proteins on autosomal gene transcription. Given that the (cytoplasmic) spermiogenesis defects are corrected in the dual mutant, it stands to reason that the secondary expression changes follow the same pattern. We therefore propose that, in addition to the nuclear effects on Group 1 genes, SLY protein has a cytoplasmic role, opposing that of SLX/SLXL1. SLY proteins have been shown to be present in both the spermatid nucleus and cytoplasm [19], [21]. Intriguingly, a recent report indicates that the acrosomal (cytoplasmic) protein DKKL1, which we previously identified as a binding partner of SLY1 [21], also interacts with SLXL1 [29]. We have performed additional experiments and observed that all SLX/Y family members (i.e. SLY1, SLY2, SLX and SLXL1) can interact with DKKL1 (Figure S7). Therefore, SLX/SLXL1 and SLY proteins could compete for interaction with (a) common partner(s) in the cytoplasm, and this competition could be at the basis of the opposite effects of SLX/SLXL1 and SLY on spermiogenesis and autosomal gene expression. A combined model proposing how SLX/SLXL1 and SLY proteins have antagonistic effects in both the spermatid nucleus and cytoplasm is presented in Figure 5.

We recognize that under our preferred model, it is difficult to explain the directionality of the expression changes seen in shSLX relative to WT, which was predominantly up-regulation of autosomal genes with comparatively few down-regulated genes [28]. A potential explanation for this lies in the spermatid developmental delay resulting in delayed spermatid elongation in shSLX. This could potentially skew the round spermatid population in shSLX testes towards earlier stages, i.e. proportionally more step 1 spermatids and fewer step 7–8 spermatids. Since there is a progressive transcriptional shutdown throughout spermatid development as chromatin is repackaged in preparation for nuclear condensation, this would thus manifest in shSLX as a selective up-regulation of those genes expressed specifically in early stage round spermatids (which in turn is plausible given the annotated functional categories for these Group 2 genes). Testing this interpretation will require further experiments on fractionated, staged sub-populations of round spermatids.

### The mouse X and Y chromosomes are involved in an intragenomic conflict that is regulated by *Slx/Slxl1* and *Sly*

Irrespective of the precise molecular mechanism(s) underlying the antagonistic effects of SLX/SLXL1 and SLY, our results demonstrate that both genes have an effect on offspring sex ratio. In particular, comparing shSLX (where *Sly* is still present) to the dual knockdown, there is a significant excess of males; and when comparing shSLY (where *Slx/Slxl1* are still present) to the dual mutant, there is a trend towards excess of females. Thus, the net effect of these genes on inheritance is for X-linked family members to favour X chromosome transmission, and Y-linked members to favour Y chromosome transmission, constituting a *prima facie* genomic conflict. Such a conflict was first postulated in the 1990s following observations that male mice with a partial deletion of the Y long arm produce an excess of female offspring, however supporting evidence has not been forthcoming until recently [16], [20], [26], [27]. The present study demonstrates that such a conflict exists between the sex chromosome-linked *Sycp3*-related genes. An intragenomic conflict is often not visible under normal conditions (i.e. in a WT population) [11], [13] and here the positive effect of *Slx/Slxl1* on sex chromosome transcription was uncovered by the production of mice that are deficient for both *Sly* and *Slx/Slxl1*; similarly, the effects of *Slx/Slxl1* deficiency are also corrected in the dual mutant, although the molecular mechanisms involved are less clear.

### Can sex ratio distortion be directly attributed to *Slx/Slxl1* and *Sly*?

Under the distorter/responder model exemplified by the t complex [1], both *Slx*/*Slxl1* and *Sly* are transmission distorters in that changes in their expression levels lead to a distortion of the sex ratio. However, it is unlikely that they are *directly* responsible for mediating the transmission skew (i.e. responder genes). Indeed, the physiological mechanism of the skew in the present model is an asymmetry in fertilizing ability between X and Y sperm [30]. This implies an underlying molecular/functional asymmetry, namely the presence of a responder gene product which is not evenly shared between X and Y sperm. Both of the known mammalian examples of transmission ratio distortion depend on non-sharing of gene products (both transcript and protein) between sister spermatids: *Spam1* in the case of Rb(6.16) and Rb(6.15) translocation heterozygotes, and *Tcr^Smok^* in the case of driving *t* haplotypes [31]–[33]. We note that SLX/Y proteins appear to be similarly expressed in X- and Y-bearing spermatids. It therefore seems likely that the distortion in Yq deleted mice and in shSLXshSLY transgenic models is mediated by an as yet unidentified sex-linked gene or gene(s) (i.e. the responder), for which *Slx/Slxl1* and *Sly* are competing regulators *via* their global effects on sex chromatin expression. Among the deregulated genes, a few appear as promising candidates, such as the X-encoded homolog of *Tcp11*, which is one of the genes involved in the t-complex transmission distortion, albeit as a distorter rather than a responder [34], and *Alkbh7*, since another *Alkbh* gene has recently been found to cause sex ratio distortion [35].

However, there may be several linked genes involved, at least one of which is likely to evade transcript sharing. In view of this possibility, it is worth noting that both regulators of the conflict have a global effect on sex chromatin; this is an efficient way to control multiple sex chromosome-linked distorters and/or responders simultaneously. The ease of identifying the responder(s) will depend on how directly *SLX*/*Y* regulate them and how many there are. Finally, it is possible that autosomal factors also contribute to the regulation of sex-linked transmission distortion. We note that historically, *Slx* appeared on the X before *Sly* appeared on the Y, and its distorting effect on sex ratio may have subsequently been countered by a combination of *Sly*-mediated repression and other autosomal genes being selected to favour a balanced sex ratio [20].

### The intragenomic conflict in which *Slx/Slxl1* and *Sly* are involved has influenced the structure of the mouse sex chromosomes

In the mouse lineage, there has been a remarkable amplification of spermatid-expressed sex chromosome genes (all of which fall into Group 1 identified above), and which has had a dramatic influence on the structure of the mouse sex chromosomes. This expansion occurred subsequent to the appearance of *Sly*, but was not accompanied by a matching increase in XY transcript levels [20]. It is therefore very likely that essential sex-linked spermatid-expressed genes have become amplified in order to maintain a steady expression in the face of the enhancement of *Sly*-mediated repression and in a sense constitute a “collateral damage” arising from the conflict between *Sly* and *Slx/Slxl1* that we unravel here. Interestingly, the *Speer* gene cluster is one of the autosomal gene families that have experienced the largest rodent-specific expansions [36] and is also repressed by SLY. *Slx/Slxl1* and *Sly* competition may therefore have led to the amplification of reproductive genes outside the sex chromosomes as well as on them.

### The intragenomic conflict in which *Slx/Slxl1* and *Sly* are involved may have played an important role in mouse speciation

**Figure 6:**
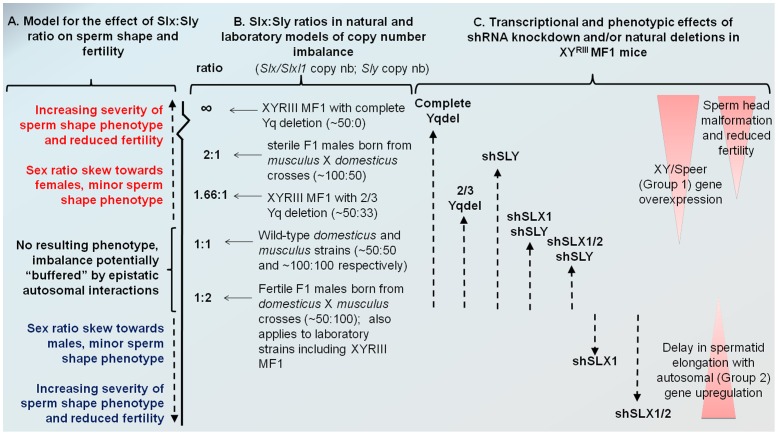
Model comparing *Slx:Sly* copy number imbalance in natural and laboratory mouse strains to *Slx:Sly* gene expression imbalance in shRNA knockdown models. A. A model for how *Slx/Slxl1*:*Sly* imbalance affects sperm shape, offspring sex ratio and fertility. B. Approximate copy number ratio of *Slx/Slxl1* and *Sly* in the reciprocal crosses studied by Good et al. [37] based on an estimate of ∼100 copies of each gene in *musculus* and ∼50 in *domesticus*, in the WT laboratory strain MF1 Y^RIII^ which has a *domesticus* X and autosomes but a *musculus*-derived Y [40]–[42], and in the two natural mutants from the same background studied by us and others [18], [20]. C. The relative magnitude of Group 1 and Group 2 transcriptional responses seen in the various shRNA/deletion models on the MF1 Y^RIII^ background. The double and triple shRNA models show a partial Group 1 response, but no Group 2 response. Importantly, in this model, the shSLX1 and shSLX1/2 phenotypes are expected to fall outside the range of variation seen in the natural mutant and reciprocal cross males, since they are on a background which has already a deficiency in *Slx/Slxl1* copy number compared to *Sly* (50∶100). We emphasise that the effects of *Slx/Slxl1*:*Sly* imbalance are only one contributor to hybrid sterility: sperm shape and testis size QTLs on the *musculus* X map to distinct locations and show different interactions with the *domesticus* autosomes and Y chromosome [50].

F1 hybrid sterile males produced by asymmetric crosses between *M. m. musculus* and *M. m. domesticus* display sperm differentiation defects and wide-spread overexpression of X-encoded spermiogenic genes [37]. Intriguingly, this only occurs in males with a *M. m. musculus* X chromosome and *M. m. domesticus* Y and autosomal chromosomes [38]. These males have an excess of *Slx/Slxl1* copies compared to *Sly* copies, since the *M. m. domesticus* X and Y chromosomes carry ∼40 to 60 copies of *Slx/Slxl1* and *Sly*, while XY encoded *Sycp3*-related genes have been more amplified in *M. m. musculus*, with >100 of *Slx/Slxl1* and *Sly* on the X and Y [18], [20]. Our data show that a balance between *Slx/Slxl1* and *Sly* expression exists in wild-type populations and that disruption of this balance can cause male infertility. In light of these data, we propose that deficiency in the number of *Sly* copies compared to *Slx/Slxl1* copies contributes to F1 male hybrid sterility (see Figure 6) in some of these crosses. This would explain the observed over-expression of X-encoded spermiogenic genes observed in some F1 hybrid males [37] and subsequent sperm differentiation defects and infertility. The observation that F1 males born from the reciprocal cross *domesticus x musculus* are reproductively normal [39] does not necessarily challenge this model. These males have an excess of *Sly* copies compared to *Slx/Slxl1* copies and, according to our model, could be considered as *Slx/Slxl1*-deficient mice and thus display some spermiogenic defects. This however depends critically on the mechanism of the antagonistic effects of SLY and SLX/SLXL1 in the cytoplasm, and on the threshold of copy number imbalance required to trigger abnormal spermatogenesis and/or sex ratio skewing. Given that autosomal genes will be selected to maintain a balanced sex ratio, the *Slx*/*Sly* conflict may well be “buffered” to some extent by epistatic interactions with autosomal genes.

We have observed that mice with a partial knockdown of *Slx/Slxl1* (shSLX1 or shSLX2) have comparatively minor spermiogenic defects compared to mice with a severe knock-down (shSLX1/2) [28]. We also note that laboratory strain X chromosomes (including MF1 mice which were used in the present study) are predominantly derived from a *domesticus* background [40], [41], yet are paired in these strains with a *musculus* Y chromosome Y^RIII^ [42]. Thus laboratory strains are intrinsically comparable to the reciprocal cross. Our shSLX models therefore involve skewing the balance of SLX/SLXL1 and SLY even further, to pathogenic effect (see Figure 6). In this light it is intriguing that WT MF1 males have lower XY gene transcription than *Slx/y* deficient males: might this reflect the fact that laboratory strains are inherently “overdosed” for *Sly* relative to *Slx/Slxl1* by virtue of their hybrid origin?

Male hybrid sterility is a complex trait involving several X-linked loci (as demonstrated by the mapping of several quantitative trait loci – QTL – on the X chromosome [38], [43], [44]) as well as autosomal factors [45], [46]). It is worth noting that among the four non-overlapping X-chromosome-linked QTL associated with abnormal spermheads and hybrid sterility, one encompasses *Slx* (0–37.1 Mb), the other, *Slxl1* (47.9–81.8 Mb) [38]. Interestingly, it has been shown that one of the autosomal loci linked to hybrid sterility, *Prdm9*, encodes a histone H3 lysine 4 methyltransferase involved in the silencing of the sex chromosomes during meiosis (Meiotic Sex Chromosome Inactivation). It therefore epigenetically represses multiple X-chromosome loci, some of which part of the hybrid sterility gene network, and epistatic interactions between *Prdm9* and multiple X and autosomal loci have been shown to cause asymmetric hybrid male sterility associated with a disruption of MSCI and thus a de-repression of the X chromosome [43], [46]. However, *Prdm9* does not appear to be involved in the X-chromosome up-regulation and sterility observed in F1 hybrid males studied by Good et al. [37].

Taken together, the genetic basis of reproductive isolation in mice is complex, and disruption of the transcriptional regulation of the X seems to contribute to the evolution of hybrid male sterility. The antagonistic effects of *Slx/Slxl1* and *Sly* at the transcriptional and phenotypic level, in particular the effects on postmeiotic XY gene regulation, may therefore be among the important elements contributing to the evolution of hybrid sterility between mouse species. The production of F1 males with a transgene-derived increased *Sly* expression or with a knockdown of *Slx/Slxl1* expression should help address this question.

In conclusion, we have demonstrated that the mouse X and Y chromosomes are involved in an intragenomic conflict that is regulated by the multicopy genes *Slx/Slxl1* and *Sly*. SLX/SLXL1 and SLY proteins compete during sperm differentiation, and notably have opposite effects on the regulation of sex chromosome gene expression. Disruption of *Slx/y* balance causes sex ratio distortion, sperm differentiation defects and male infertility. To the best of our knowledge, our work is the first characterization of a conflict over sex chromosome transmission in mammals and provides further evidence to support the hypothesis that intragenomic conflicts can have major consequences on gene regulation, genome structure and speciation.

## Materials and Methods

### Generation and breeding of transgenic mice

shSLY (aka sh367), shSLX1 and shSLX1/2 males were produced and maintained as described before [19], [28]. To produce shSLX1shSLY and shSLX1/2shSLY double transgenic mice, shSLX1 females were mated to shSLY or to shSLYshSLX2 transgenic males. Double transgenic females were then mated to MF1 XY^RIII^ males (see [19]) to maintain the stock, since shSLY males are subfertile and give progeny only rarely. Two-month-old males single or double transgenic for sh367 (shSLY), shSLX1 or shSLX1/2 transgenes, as well as their non-transgenic siblings (WT) were processed for all the analyses presented here. Animal procedures were in accordance with the United Kingdom Animal Scientific Procedures Act 1986 and were subject to local ethical review.

### Elutriation of spermatids

Fractions enriched in round spermatids (>90%) were obtained from the above described transgenic and control (WT) males as described previously [19]. Each sample has been purified from a pool of testes obtained from 2 to 5 males.

### Transfection

The coding sequence of mouse *Dkkl1* and *Slx* cDNA were amplified by PCR and cloned into a C-terminal Myc-tagged pCMV vector; the coding sequence of mouse *Slx*, *Slxl1*, *Sly1* and *Sly2* cDNA were amplified by PCR and cloned into a N-terminal Flag tagged pCMV vector using EcoRI and NotI restriction sites (see Table S1 for a full list of primers). Co-transfections of HEK293 or COS cells were performed in 6-well plates using 1.5 µg of each DNA and 5 µl of Lifofectamine (Invitrogen) following the manufacturer's instructions. Proteins were extracted 24 hours post transfection in 200 µl of Lysis buffer (25 mM NaCl, 10 mM Tris-HCl, 5 mM EDTA, 0.1%NP-40) and immunoprecipitated as described below.

### Protein analyses

Nuclear and cytoplasmic protein extracts were obtained as follow. The powder obtained from two adult testes crushed on dry ice was homogenized in a glass pestle with 1 mL of lysis buffer (0.6 M Sucrose, 10 mM Hepes pH 7.7, 25 mM KCl, 2 mM EDTA, 0.5 mM EGTA and protease inhibitors). After the addition of 0.2% NP40, the lysate was centrifuged for 15 minutes at 800 g. The supernatant corresponded to the cytoplasmic fraction. The pellet was washed twice with 1 mL of lysis buffer and then resuspended in 100 µl of nuclear protein extraction buffer (20 mM Hepes pH 7.7, 1.5 mM MgCl2, 0.2 mM EDTA, 25% glycerol and protease inhibitors) plus 10 µl of 4 M NaCl. After 30 minutes of homogenization at 4°C, the samples were centrifuged for 30 minutes at 11000 g; the supernatant corresponded to the nuclear protein extract. A pellet of ∼1×10^7^ round spermatids was extracted following the same protocol using 250 µl of lysis buffer and 50 µl of nuclear protein extraction buffer. Whole testicular protein extraction was performed as described previously [19]. For immunoprecipitation experiments, proteins extracted from transfected cells were first pre-cleared with protein A/G sepharose for 1 hour at 4°C. They were then incubated overnight with Protein G- or Protein A- sepharose which had been covalently bound to MYC (Santa Cruz Biotechnology) or FLAG (Sigma) antibody beforehand (see [21] for a detailed protocol). Western blot experiments were performed as described previously [19]. Membranes were incubated overnight with anti-SLX/SLXL1 [28] diluted at 1/3000, anti-SLY1 [21] at 1/3000, anti–β-actin (Sigma) at 1/50000, or anti-LAMIN B1 (Santa Cruz Biotechnology) at 1/1000, anti-FLAG (Sigma) at 1/1000, or anti-MYC (Santa Cruz Biotechnology) at 1/1000. Detection by chemiluminescence was carried out after incubation with the corresponding secondary antibody coupled to peroxidase, as described by the manufacturer (Millipore).

### Immunofluorescence and TUNEL

Immunofluorescence experiments were performed on testis material fixed in 4% buffered paraformaldehyde and sectioned as described before [25]. DAPI (4′,6-diamidino-2-phenylindole) was used to stain nuclei (Vectashield DAPI, Vectorlab). Alexa Fluor 594-conjugated peanut agglutinin lectin (Invitrogen), which stains the developing acrosome of spermatids, was used to stage the testis tubules. For the analysis of apoptotic elongating spermatids and delayed elongating spermatids, approximately 150 tubules were counted per individual (4 to 6 individuals per genotype). The percentage of tubules containing apoptotic elongating spermatids was determined on testis sections fluorescently stained using an in situ cell death detection kit (TUNEL, terminal deoxynucleotidyltransferase dUTP nick end labeling) as described by the manufacturer (Roche Diagnostics, Indianapolis, IN). The percentage of tubules containing delayed elongating spermatids (i.e. stage I to VIII tubules containing elongating spermatids) was assessed on testis sections fluorescently stained by H4K12Ac antibody (Millipore, Bedford, MA), a known marker of stage 9–12 elongating spermatids.

### Antibody detection, chromosome painting, and DNA–FISH on surface-spread testicular cells

Antibody detection was performed on surface-spread testicular cells following a protocol described previously [19] adapted from Barlow et al. [47]. Incubation with the primary antibody (anti-SLY1 [17], anti-SLX/SLXL1 [28] or anti-H3K9me3 [Upstate] diluted 1/100) was carried out over-night in a humid chamber at 37°C. DNA-FISH, then chromosome painting were performed after antibody detection as described previously [48]. *Speer* DNA-FISH was carried out using mouse BACs RP23-212A20 and RP24-310N20 (CHORI). As a control for specificity (see Figure S1), SLX/SLXL1 antibody was preabsorbed with 8 mg of SLX immunogenic peptide or with 8 mg of a noncompeting peptide (SLY peptide). For the quantification of H3K9me3 signal over the PMSC, the chromocenter domain was defined using the corresponding black and white DAPI picture. Then, H3K9me3 signal outside this chromocenter domain was measured and normalized to that of H3K9me3 signal over the chromocenter for each cell (100 cells per genotype), using Metamorph and ImageJ (See Figure S4). Slides corresponding to 3 individuals per genotype were coded and randomized before the analysis; the analysis was therefore carried out blind as to genotype.

### Analysis of sperm head morphology

For the quantification of spermhead abnormalities, sperm collected from the initial caput epididymis were suspended in phosphate-buffered saline. The suspension was smeared on slides (two slides per individual) and fixed in 3∶1 methanol∶acetic acid. The slides were then dipped in 0.4% Photoflo for 2 min, air dried and stained on a plate heated at 60°C with one drop of 50% silver nitrate mixed with one drop of 2% gelatin (Sigma). The slides were coded and randomized. Sperm scoring was carried out ‘blind’ as to genotype (4 to 6 individuals per genotype) and 100 sperm per slide were classified into 6 categories on the basis of the type and severity of abnormalities observed, using criteria described by Yamauchi et al. [49] and in Figure S6. In the text and figures, spermheads from category N were termed “normal”; category 1S, “slightly abnormal”; category 2S, “slightly flattened”; category 3G, “abnormally thin”; category 4G, “grossly flattened” and categories 5G to 8G were pooled and named “other gross abnormalities” (cf. Figure S6).

### Fertility testing and sex ratio of the offspring

To assess fertility and obtain sex ratio data from the offspring, five males of each genotype were mated with MF1 WT females over a period of six months.

### Real-time PCR and microarray analyses

Real-time Reverse Transcription-Polymerase Chain Reaction (RT-PCR) and microarray analyses were performed as previously described on RNA extracted from 2-month old testis or from round spermatids obtained after elutriation [19] (cf. Table S1 for a list of the primers used in this study). Real-time RT-PCR experiments were performed in parallel for all the genotypes described in this study, with between 3 to 5 individuals per genotype. For the microarray analysis, three shSLX1, three shSLY, three shSLX1shSLY and four wild type spermatid batches were analyzed (Illumina BeadChip, mouse whole-genome array, v2). These data thus include and extend our previously-reported results for shSLX1 round spermatids and for shSLY round spermatids in previous analyses [19], [28], which collectively used two shSLY, two shSLX1 and four WT spermatid batches. Data normalization and calculation of FDR-adjusted p values was carried out in BeadStudio (Illumina) as previously described [19], [28]. The full data set has been uploaded to GEO, accession number GSE39109.

### Statistical analysis

For comparisons of the incidence of sperm head abnormalities, differences between genotypes were assessed by ANOVA after angular transformation of percentages, using the General Linear Models ANOVA provided by NCSS statistical data analysis software. The same test was applied to the frequency of abnormal head-tail connections, TUNEL positive elongating spermatids, delayed elongating spermatids (assessed by H4K12Ac staining) and H3K9me3 quantification. Student's *t* test was used to compare the data obtained for fecundity, sperm number and real-time PCR (performed on the ΔCt values). A Chi-square test was used for sex ratio data. Microarray results were analyzed as described in Figure S3 and Figure S5.

## Supporting Information

Figure S1 Immunofluorescence detection of SLX/SLXL1 proteins in spermatids. A) Representative pictures of the detection of SLX/SLXL1 proteins (green) by immunofluorescence in shSLY and WT round spermatid nuclei (surface spread technique). DAPI (blue) was used to stain nuclei. X and Y chromosome painting were performed sequentially. SLX/SLXL1 proteins are detected in shSLY spermatid nuclei in 76% of the cases. No signal could be detected in the majority of WT round spermatid nuclei (84%). The nuclear SLX/SLXL1 signal observed in the remaining ∼16% of WT round spermatid is very weak compared to the nuclear signal in shSLY round spermatids. B) Control of the specificity of SLX/SLXL1 immunofluorescence signal (green) in surface-spread round spermatid nuclei. DAPI (in blue) was used to stain nuclei. Left Panel: SLX/SLXL1 proteins (in green) were observed in *Sly*-deficient (shSLY) round spermatid nuclei. Note in the picture the presence of a flattened sperm head, characteristic of shSLY testicular spread. When the antibody was preabsorbed with SLX/SLXL1 peptide, the signal disappeared. When the antibody was preabsorbed with a noncompeting peptide (SLY), SLX/SLXL1 signal was maintained. Right Panel: No signal was observed in the majority of WT round spermatids, in round spermatids deficient for SLX/SLXL1 proteins (shSLX1/2) and those deficient for both SLY and SLX/SLXL1 (shSLX1/2shSLY). All these controls demonstrate the specificity of the nuclear signal obtained with SLX/SLXL1 antibody in shSLY round spermatids.
(TIF)

Figure S2 Characterization of shSLX1/2shSLY males. We produced males carrying an shSLY transgene together with one or two shSLX transgenes (i.e. shSLX1 and/or shSLX2). ShSLY males carry an shRNA-expressing transgene, which triggers the specific degradation of *Sly* transcripts *via* RNA interference [19]. Similarly, shSLX transgenic mice express *Slx/Slxl1*-specific shRNA and display a decrease in whole testis *Slx/Slxl1* transcript levels estimated as ∼68% and ∼59% for transgenic lines shSLX1 and shSLX2, and ∼83% for shSLX1/2 double transgenics [28]. A–B) Real time PCR quantification of *Slx*/*Slxl1* (A) and *Sly* (B) transcript levels in WT, shSLY, shSLX2shSLY, shSLX1shSLY and shSLX1/2shSLY whole testes. The y-axis indicates the level of expression compared to WT (2^ΔΔCt^ ± standard errors). The combination of shSLY transgene with any shSLX transgene yielded an efficient knockdown of *Sly* and of *Slx/Slxl1* [one asterisk indicates significant difference from WT (p<0.02; t test on ΔΔCt values)]. The combination of shSLY transgene and two shSLX transgenes (i.e. shSLX1/2shSLY) produced a more pronounced decrease in *Slx/Slxl1* expression [two asterisks indicate significant difference between shSLX1/2shSLY and shSLX2shSLY or shSLX1shSLY (p<0.02; t test on ΔΔCt values)]. C) Real time PCR quantification showed that transcript levels of X-encoded (*Actrt1* and *1700008I05Rik*) and of Y-encoded genes (*Ssty1* and *Zfy2*) were lower in shSLX1/2shSLY than in shSLY whole testes [one asterisk indicates significant difference between shSLY and shSLX1/2shSLY values (p<0.05; t test on ΔΔCt values)]. The y-axis indicates the level of expression compared to WT (2^ΔΔCt^ ± standard errors).
(TIF)

Figure S3 Comparison of the microarray results obtained for shSLY and shSLX1shSLY round spermatids. List of the 222 genes showing greater than 1.5 fold-change in *Sly*-deficient spermatids (i.e. shSLY) relative to WT; 196 of them were corrected to some degree by addition of the shSLX transgene (in shSLX1shSLY). For 46 of these genes, the difference between shSLY and shSLX1shSLY was itself statistically significant.
(PDF)

Figure S4 Measurement of the intensity of H3K9me3 staining over PMSC in surface-spread spermatids. (A) Graph representing the distribution of the H3K9me3 PMSC signal intensity per spermatid as classified by categories in WT (red), *Sly*-deficient (shSLY, blue) and *Slx/y*-deficient mice (shSLX1/2shSLY, green). The average values obtained for WT, shSLY and shSLX1/2shSLY are respectively: 0.65, 0.51 and 0.59. B) Graph representing the H3K9me3 PMSC signal intensity in each spermatid, ranked by increasing intensity, in WT (red), *Sly*-deficient (shSLY, blue) and *Slx/y*-deficient mice (shSLX1/2shSLY, green). The median values obtained for WT, shSLY and shSLX1/2shSLY are respectively: 0.64, 0.52 and 0.58.
(TIF)

Figure S5 Comparison of the microarray results obtained for shSLX1 and shSLX1shSLY round spermatids. List of the 115 genes showing greater than 1.5 fold-change in *Slx/Slxl1*-deficient spermatids (i.e. shSLX1) relative to WT; 111 of them were corrected to some degree by addition of the shSLY transgene (in shSLX1shSLY). For 91 of these genes, the difference between shSLX1 and shSLX1shSLY was itself statistically significant.
(PDF)

Figure S6 Further analyses of the sperm morphology of *Slx/y*-deficient males. A) Bar graph representing the percentage of spermatozoa with abnormal head to tail connections. *Slx/Slxl1*-deficient spermatozoa (i.e. shSLX1 or shSLX2) displayed a significant increase of abnormal head to tail connections compared to WT (represented by the hatch symbol, p<0.001; ANOVA). This was significantly rescued by the addition of shSLY transgene with values from shSLX1shSLY, shSLX2shSLY and shSLX1/2shSLY males undistinguishable from WT (ANOVA, p>0.4) but significantly different from shSLX1 and shSLX2 values (represented by a star; ANOVA, p<0.05). B) Pictures and diagrams of the different categories of sperm heads used for the quantification, as previously described by Yamauchi et al. [49]. N: normal sperm. Category 1S: slightly abnormal sperm heads with changes in the caudal part. Category 2S: heads with slightly flattened acrosome. Category 3G: heads which are thinner and more pointed. Category 4G: heads with grossly flattened acrosome (more severe version of 2S). Categories 5G to 8G were pooled and represent other gross abnormalities. 5G: head-neck junction located on the ventral side. 6G–8G: heads without the curve normally observed in sperm, with remnants of the hook (6G) or with an oval shape (7G and 8G). Scale bar represents 5 µm. C) Detailed quantification of sperm head abnormalities in all the genotypes analyzed in the present study. D) Quantification of normal spermheads. Hatch symbol (#) indicates significant difference from WT (ANOVA, p<0.0001). One asterisk indicates significant improvement between other genotypes (ANOVA, p<0.02). Note the increase in the percentage of normal spermheads in *Slx/y*-deficient (shSLX1shSLY, shSLX2shSLY and shSLX1/2shSLY) compared to *Sly*-deficient male (shSLY). ShSLX1/2shSLY males also show a significant increase in the percentage of normal spermheads compared to shSLX1shSLY and shSLX2shSLY males. E) Quantification of grossly flattened spermhead abnormalities. These abnormalities are specifically observed in *Sly*-deficient males. Hatch symbol (#) indicates significant difference from WT (ANOVA, p<0.0001). One asterisk indicates significant improvement between other genotypes (ANOVA, p<0.03). Note the decrease in the percentage of grossly flattened spermheads in *Slx/y*-deficient (shSLX1shSLY, shSLX2shSLY and shSLX1/2shSLY) compared to *Sly*-deficient males (shSLY). ShSLX1/2shSLY males also show a significant decrease in this percentage compared to shSLX1shSLY and shSLX2shSLY males. According to those results, the genotypes can be classified in order of decreasing severity of their spermhead phenotype, as follow: shSLY>shSLX1shSLY = shSLX2shSLY>shSLX1/2shSLY. This is in clear correlation with the intensity of *Slx/Slxl1* knock-down and thus demonstrates that *Slx/Slxl1* knockdown rescues *Sly*-deficiency. F) Quantification of slightly abnormal spermhead abnormalities. These abnormalities are specifically observed in *Slx/Slxl1*-deficient males. Hatch symbol (#) indicates significant difference from WT (ANOVA, p<0.04). One asterisk indicates significant difference between *Slx/y*-deficient and *Slx/Slxl1*-deficient males (ANOVA, p<0.0008). Note the decrease in the percentage of slightly thin spermheads in *Slx/y*-deficient (shSLX1shSLY, shSLX2shSLY and shSLX1/2shSLY) compared to *Slx/Slxl1*-deficient males (shSLX1 or shSLX2). For this category of sperm abnormalities, *Slx/y*-deficient males did not significantly differ from WT. This shows that *Sly* deficiency rescues the spermhead abnormalities observed in *Slx/Slxl1*-deficient males.
(TIF)

Figure S7 SLX, SLXL1, SLY1, and SLY2 proteins can interact with the cytoplasmic protein DKKL1. A) FLAG antibody detection of extracts from COS cells transfected with DKKL1-MYC and either FLAG-SLY1, FLAG-SLY2, FLAG-SLX, FLAG-SLXL1, before (INPUT) and after immunoprecipitation (IP) with MYC antibody. A non-specific FLAG-tagged control was not immunoprecipitated by DKKL1-MYC (data not shown).
(TIF)

Table S1 List of the primers used in the study.
(PDF)
