# Correction: A Genetic Basis for a Postmeiotic X Versus Y Chromosome Intragenomic Conflict in the Mouse

**DOI:** 10.1371/journal.pgen.1008290

**Published:** 2019-07-22

**Authors:** Julie Cocquet, Peter J. I. Ellis, Shantha K. Mahadevaiah, Nabeel A. Affara, Daniel Vaiman, Paul S. Burgoyne

It has been brought to our attention that a clarification is required regarding the bottom two panels of Fig 1B and the middle 4 lanes of Fig 2E. The same images were purposefully used as comparisons for different experiments. Please view the updated Fig 2 legend here.

**Fig 2:**
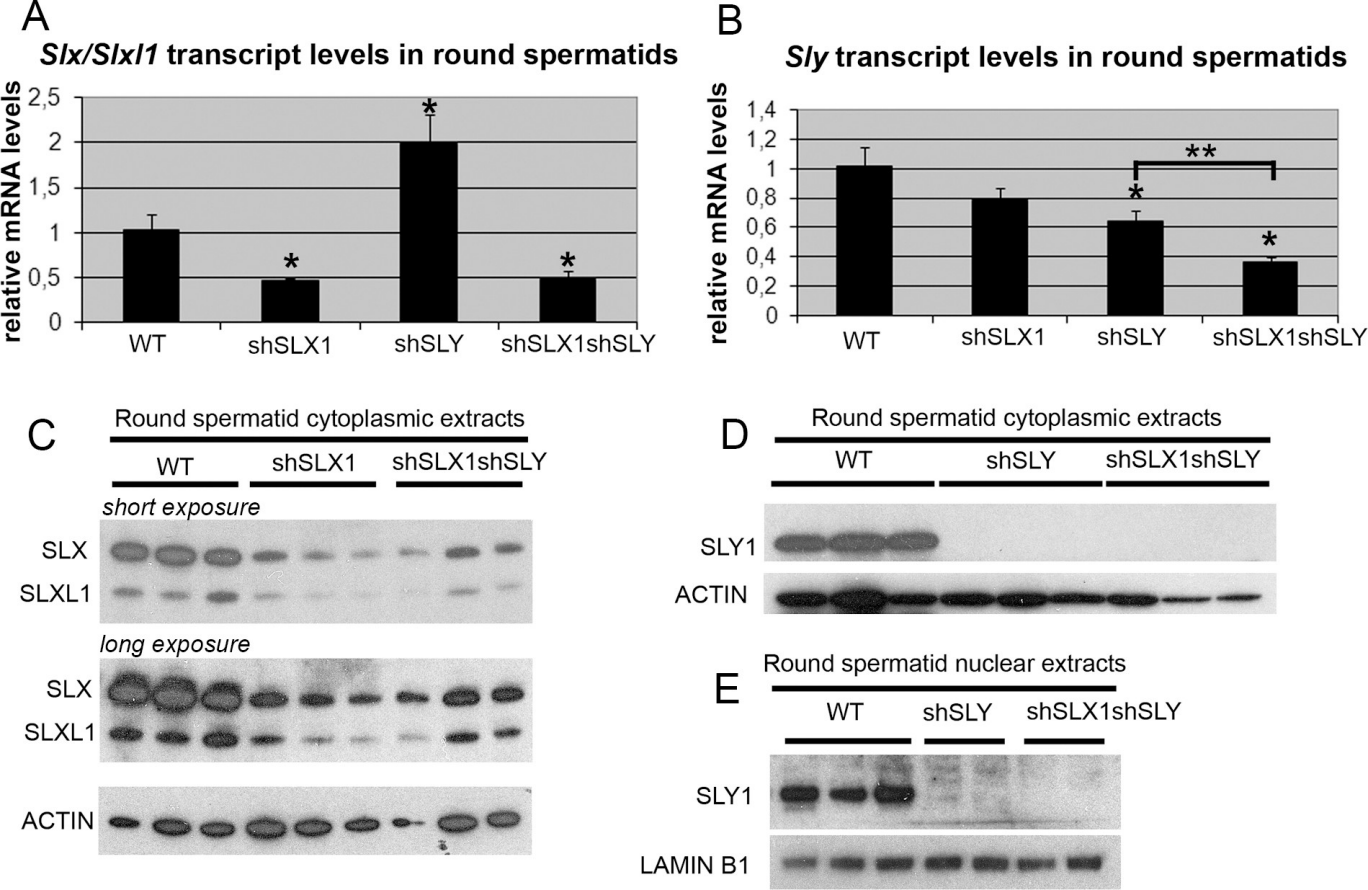
The combination of shSLX and shSLY transgenes produces an efficient knockdown of *Slx/Slxl1* and *Sly* genes. A–B) Real time PCR quantification of *Slx/Slxl1* (*Slx-all* primers) (A) and *Sly* (*Sly1* and *Sly2* variants) (B) transcript levels in WT, shSLX1, shSLY and shSLX1shSLY round spermatids. The y-axis indicates the level of expression compared to WT after normalization with *Acrv1* (2^ΔΔCt^ ± standard errors). The reduction in *Slx/Slxl1* transcript level was similar in shSLX1shSLY males and in shSLX1 siblings. As observed before [19], *Slx/Slxl1* transcript level was found increased in shSLY males. One asterisk indicates significant difference from WT (p<0.05; t test on ΔΔCt values). *Sly* knockdown was even stronger in shSLX1shSLY males compared to shSLY siblings [two asterisks indicate significant difference between shSLX1shSLY and shSLY (p  =  0.02; t test on ΔΔCt values)]. C–E) Western blot detection of SLY1, SLX and SLXL1 proteins in nuclear and cytoplasmic fractions from WT, shSLY, shSLX1 and shSLX1shSLY round spermatids. LAMIN B1 and ACTIN detection were used as loading controls for nuclear and cytoplasmic fractions, respectively. No SLY1 protein could be detected in shSLY or in shSLX1shSLY samples. For ease of comparison, the middle 4 lanes of panel Fig 2E are the same as those displayed in Fig 1B.
