# Supplementary figures and images for: A Genetic Basis for a Postmeiotic X Versus Y Chromosome Intragenomic Conflict in the Mouse
Source: PLoS Genet. 2012 Sep 13;8(9):e1002900. doi: 10.1371/journal.pgen.1002900 (PMC3441658; doi:10.1371/journal.pgen.1002900)

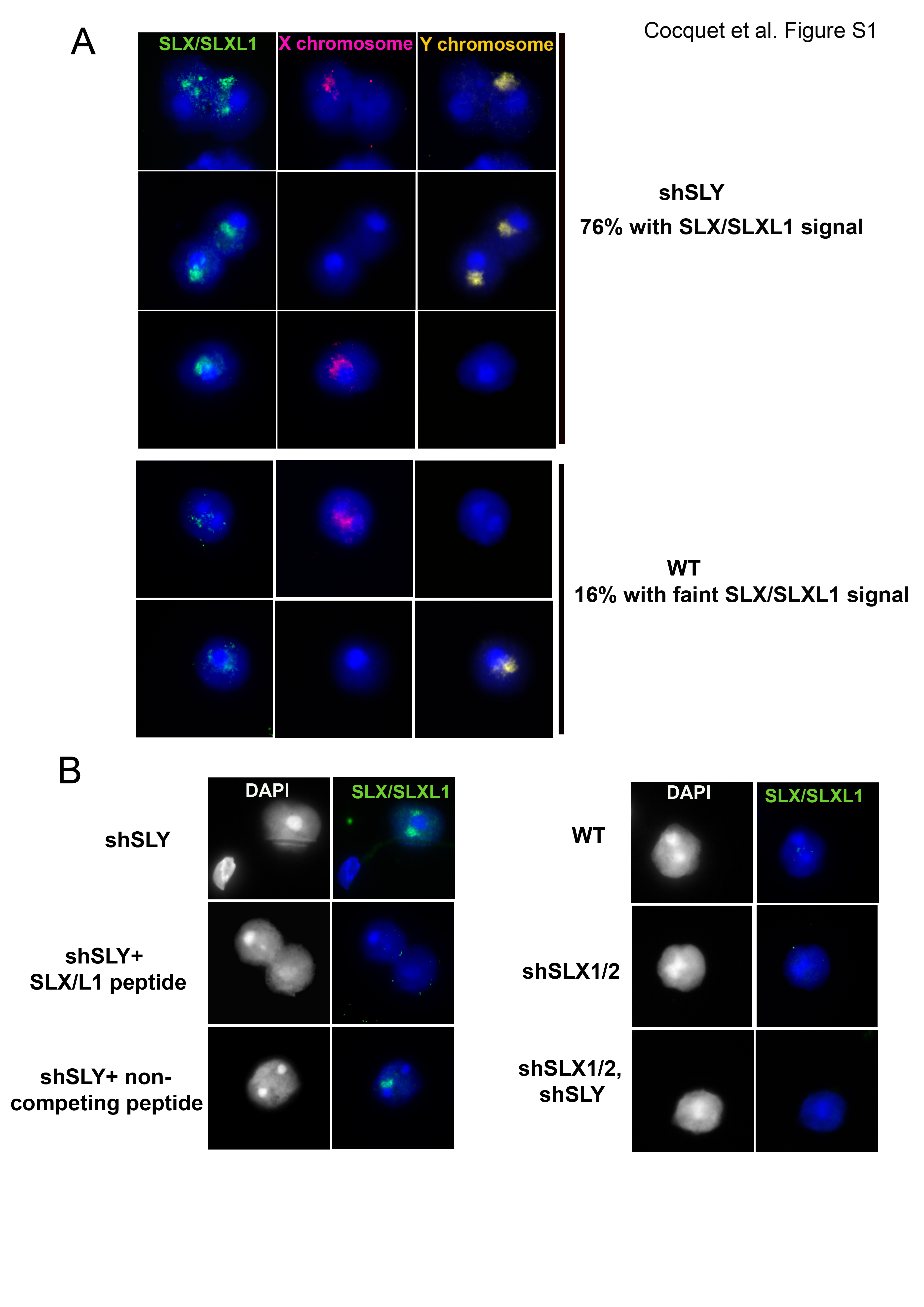

Supplement: Figure S1 — Immunofluorescence detection of SLX/SLXL1 proteins in spermatids. A) Representative pictures of the detection of SLX/SLXL1 proteins (green) by immunofluorescence in shSLY and WT round spermatid nuclei (surface spread technique). DAPI (blue) was used to stain nuclei. X and Y chromosome painting were performed sequentially. SLX/SLXL1 proteins are detected in shSLY spermatid nuclei in 76% of the cases. No signal could be detected in the majority of WT round spermatid nuclei (84%). The nuclear SLX/SLXL1 signal observed in the remaining ∼16% of WT round spermatid is very weak compared to the nuclear signal in shSLY round spermatids. B) Control of the specificity of SLX/SLXL1 immunofluorescence signal (green) in surface-spread round spermatid nuclei. DAPI (in blue) was used to stain nuclei. Left Panel: SLX/SLXL1 proteins (in green) were observed in Sly-deficient (shSLY) round spermatid nuclei. Note in the picture the presence of a flattened sperm head, characteristic of shSLY testicular spread. When the antibody was preabsorbed with SLX/SLXL1 peptide, the signal disappeared. When the antibody was preabsorbed with a noncompeting peptide (SLY), SLX/SLXL1 signal was maintained. Right Panel: No signal was observed in the majority of WT round spermatids, in round spermatids deficient for SLX/SLXL1 proteins (shSLX1/2) and those deficient for both SLY and SLX/SLXL1 (shSLX1/2shSLY). All these controls demonstrate the specificity of the nuclear signal obtained with SLX/SLXL1 antibody in shSLY round spermatids. (TIF) [file pgen.1002900.s001.tif]

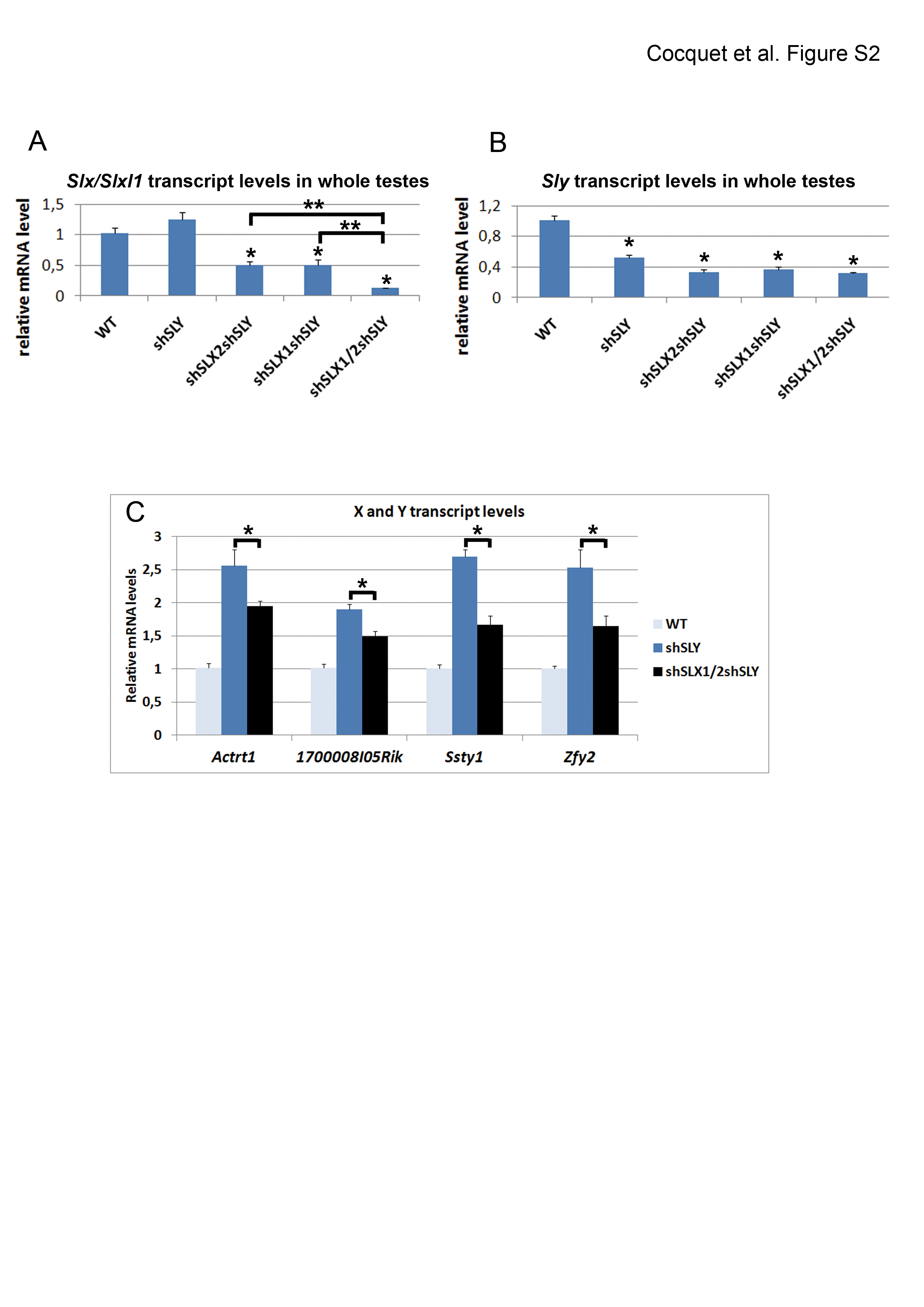

Supplement: Figure S2 — Characterization of shSLX1/2shSLY males. We produced males carrying an shSLY transgene together with one or two shSLX transgenes (i.e. shSLX1 and/or shSLX2). ShSLY males carry an shRNA-expressing transgene, which triggers the specific degradation of Sly transcripts via RNA interference [19]. Similarly, shSLX transgenic mice express Slx/Slxl1-specific shRNA and display a decrease in whole testis Slx/Slxl1 transcript levels estimated as ∼68% and ∼59% for transgenic lines shSLX1 and shSLX2, and ∼83% for shSLX1/2 double transgenics [28]. A–B) Real time PCR quantification of Slx/Slxl1 (A) and Sly (B) transcript levels in WT, shSLY, shSLX2shSLY, shSLX1shSLY and shSLX1/2shSLY whole testes. The y-axis indicates the level of expression compared to WT (2ΔΔCt ± standard errors). The combination of shSLY transgene with any shSLX transgene yielded an efficient knockdown of Sly and of Slx/Slxl1 [one asterisk indicates significant difference from WT (p<0.02; t test on ΔΔCt values)]. The combination of shSLY transgene and two shSLX transgenes (i.e. shSLX1/2shSLY) produced a more pronounced decrease in Slx/Slxl1 expression [two asterisks indicate significant difference between shSLX1/2shSLY and shSLX2shSLY or shSLX1shSLY (p<0.02; t test on ΔΔCt values)]. C) Real time PCR quantification showed that transcript levels of X-encoded (Actrt1 and 1700008I05Rik) and of Y-encoded genes (Ssty1 and Zfy2) were lower in shSLX1/2shSLY than in shSLY whole testes [one asterisk indicates significant difference between shSLY and shSLX1/2shSLY values (p<0.05; t test on ΔΔCt values)]. The y-axis indicates the level of expression compared to WT (2ΔΔCt ± standard errors). (TIF) [file pgen.1002900.s002.tif]

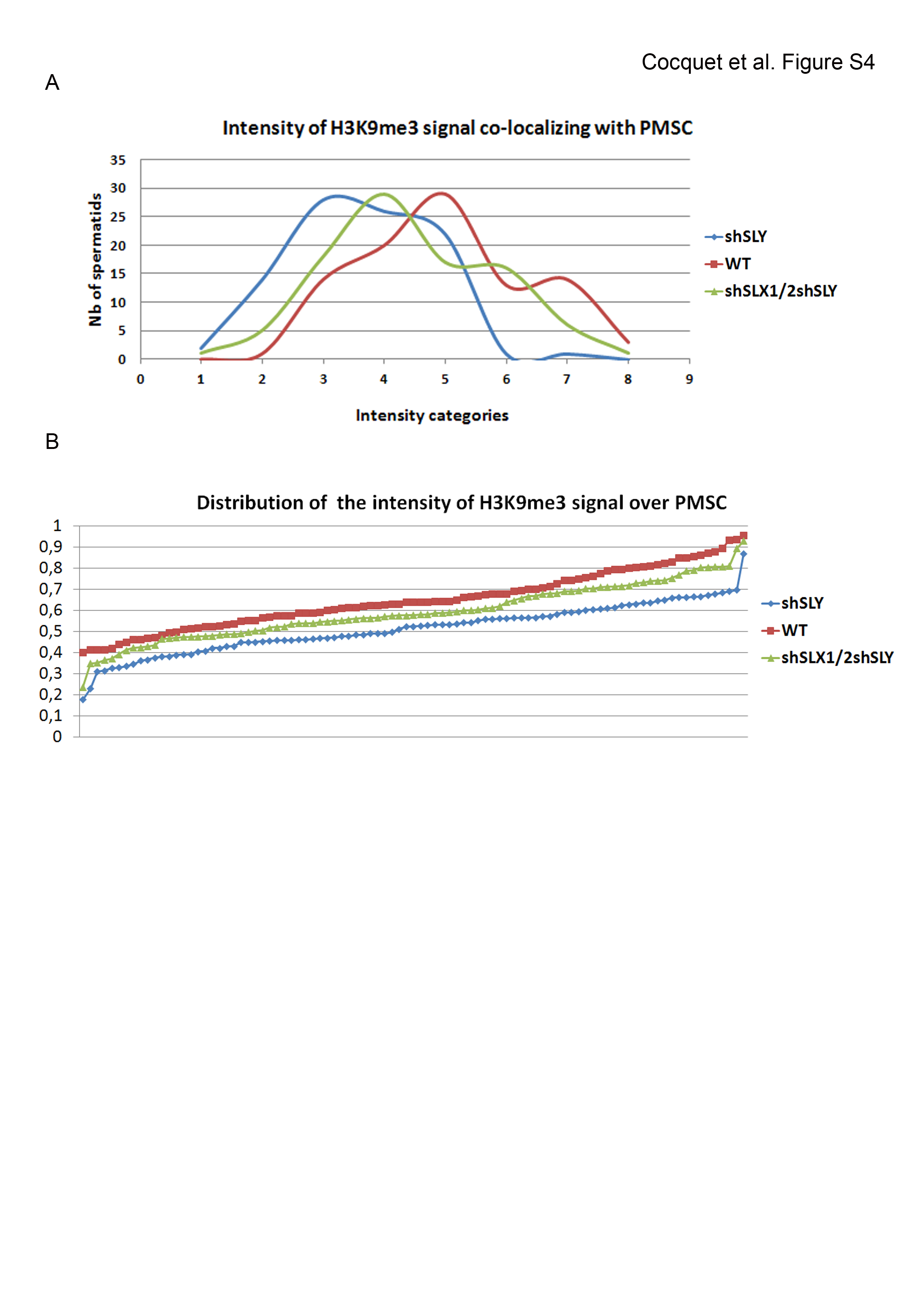

Supplement: Figure S4 — Measurement of the intensity of H3K9me3 staining over PMSC in surface-spread spermatids. (A) Graph representing the distribution of the H3K9me3 PMSC signal intensity per spermatid as classified by categories in WT (red), Sly-deficient (shSLY, blue) and Slx/y-deficient mice (shSLX1/2shSLY, green). The average values obtained for WT, shSLY and shSLX1/2shSLY are respectively: 0.65, 0.51 and 0.59. B) Graph representing the H3K9me3 PMSC signal intensity in each spermatid, ranked by increasing intensity, in WT (red), Sly-deficient (shSLY, blue) and Slx/y-deficient mice (shSLX1/2shSLY, green). The median values obtained for WT, shSLY and shSLX1/2shSLY are respectively: 0.64, 0.52 and 0.58. (TIF) [file pgen.1002900.s004.tif]

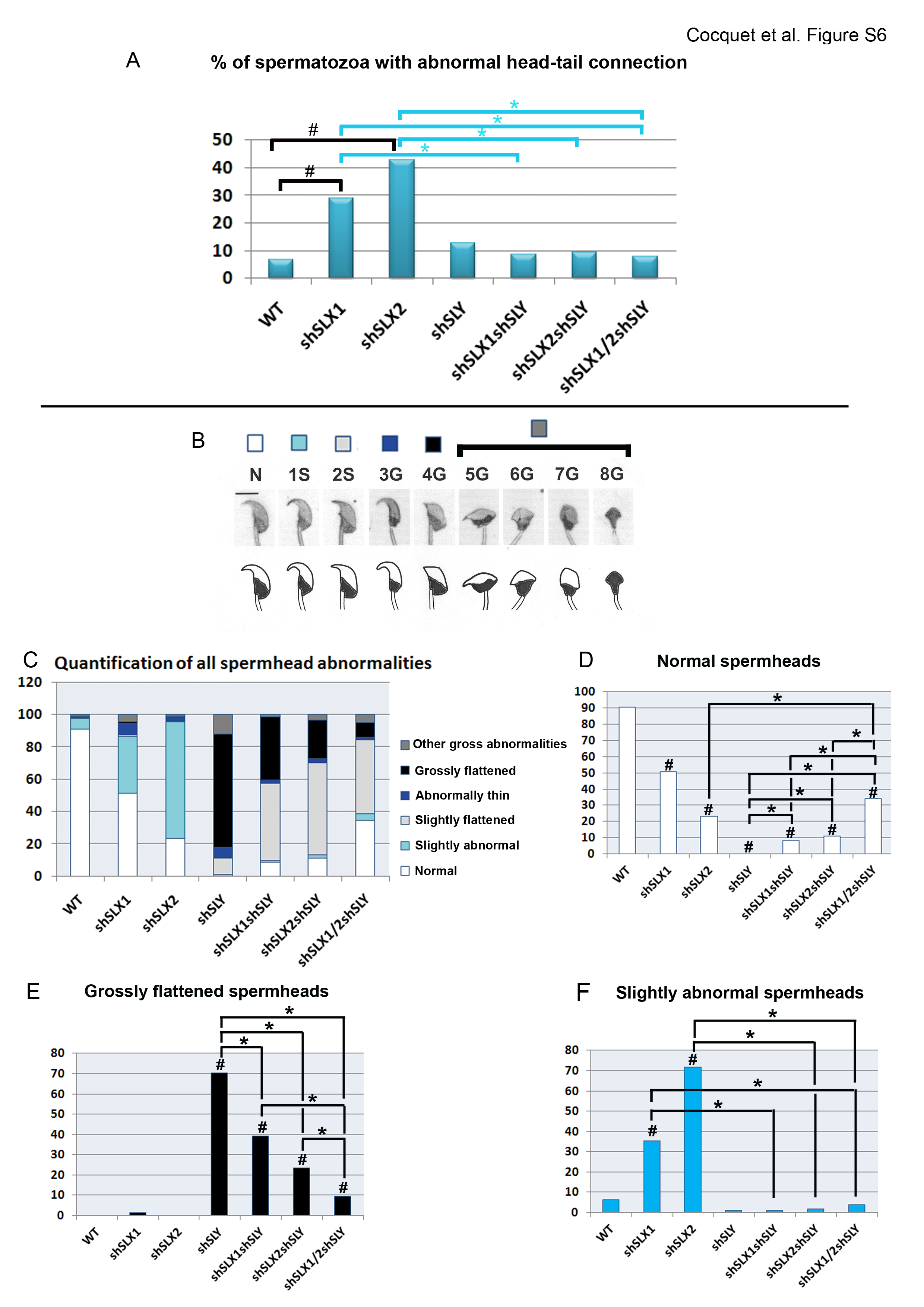

Supplement: Figure S6 — Further analyses of the sperm morphology of Slx/y-deficient males. A) Bar graph representing the percentage of spermatozoa with abnormal head to tail connections. Slx/Slxl1-deficient spermatozoa (i.e. shSLX1 or shSLX2) displayed a significant increase of abnormal head to tail connections compared to WT (represented by the hatch symbol, p<0.001; ANOVA). This was significantly rescued by the addition of shSLY transgene with values from shSLX1shSLY, shSLX2shSLY and shSLX1/2shSLY males undistinguishable from WT (ANOVA, p>0.4) but significantly different from shSLX1 and shSLX2 values (represented by a star; ANOVA, p<0.05). B) Pictures and diagrams of the different categories of sperm heads used for the quantification, as previously described by Yamauchi et al. [49]. N: normal sperm. Category 1S: slightly abnormal sperm heads with changes in the caudal part. Category 2S: heads with slightly flattened acrosome. Category 3G: heads which are thinner and more pointed. Category 4G: heads with grossly flattened acrosome (more severe version of 2S). Categories 5G to 8G were pooled and represent other gross abnormalities. 5G: head-neck junction located on the ventral side. 6G–8G: heads without the curve normally observed in sperm, with remnants of the hook (6G) or with an oval shape (7G and 8G). Scale bar represents 5 µm. C) Detailed quantification of sperm head abnormalities in all the genotypes analyzed in the present study. D) Quantification of normal spermheads. Hatch symbol (#) indicates significant difference from WT (ANOVA, p<0.0001). One asterisk indicates significant improvement between other genotypes (ANOVA, p<0.02). Note the increase in the percentage of normal spermheads in Slx/y-deficient (shSLX1shSLY, shSLX2shSLY and shSLX1/2shSLY) compared to Sly-deficient male (shSLY). ShSLX1/2shSLY males also show a significant increase in the percentage of normal spermheads compared to shSLX1shSLY and shSLX2shSLY males. E) Quantification of grossly flattened spermhead abnormali [file pgen.1002900.s006.tif]

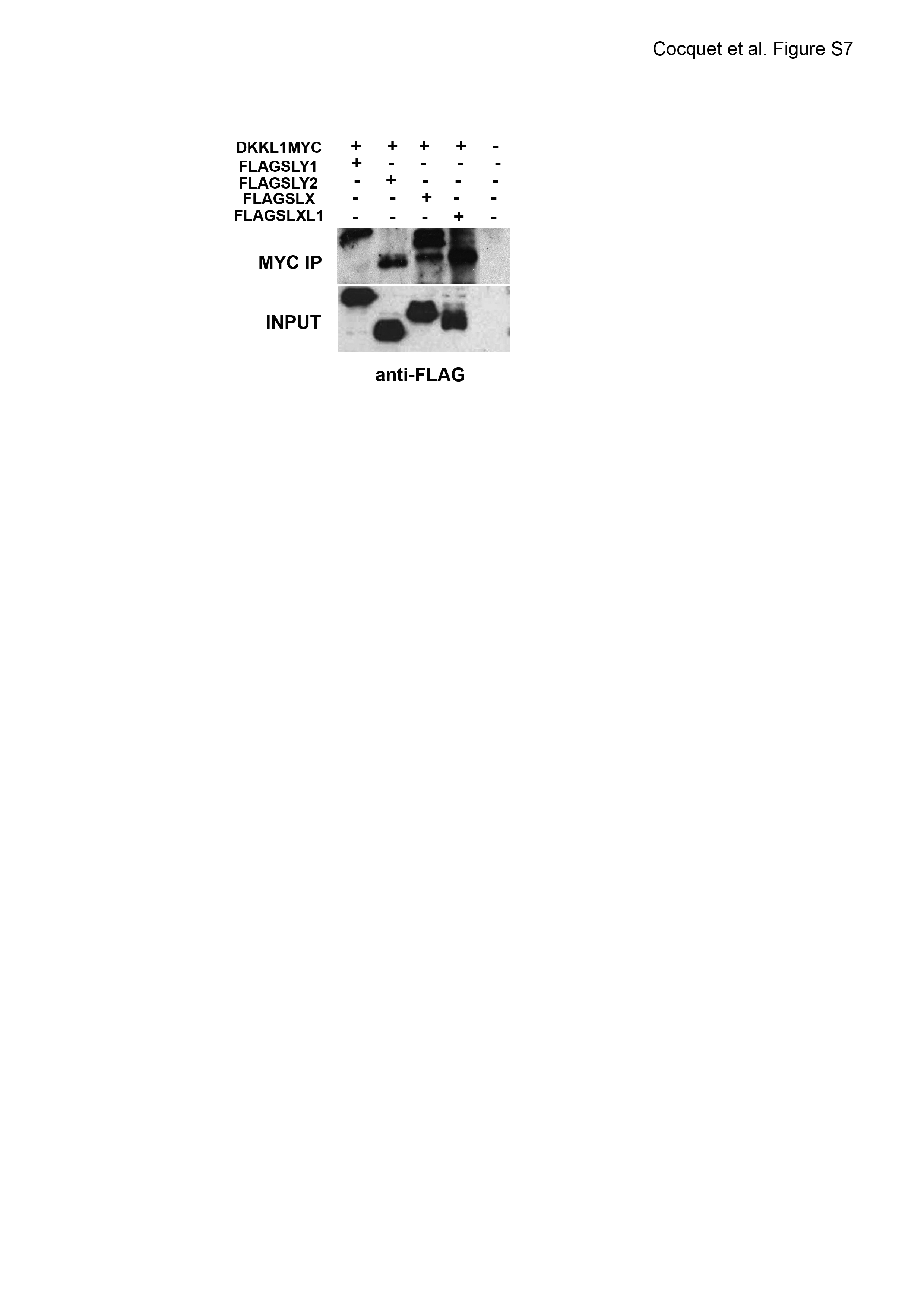

Supplement: Figure S7 — SLX, SLXL1, SLY1, and SLY2 proteins can interact with the cytoplasmic protein DKKL1. A) FLAG antibody detection of extracts from COS cells transfected with DKKL1-MYC and either FLAG-SLY1, FLAG-SLY2, FLAG-SLX, FLAG-SLXL1, before (INPUT) and after immunoprecipitation (IP) with MYC antibody. A non-specific FLAG-tagged control was not immunoprecipitated by DKKL1-MYC (data not shown). (TIF) [file pgen.1002900.s007.tif]
