# Supplementary material for: A Genetic Basis for a Postmeiotic X Versus Y Chromosome Intragenomic Conflict in the Mouse
Source: PLoS Genet. 2012 Sep 13;8(9):e1002900. doi: 10.1371/journal.pgen.1002900 (PMC3441658; doi:10.1371/journal.pgen.1002900)
Supplement: Figure S3 — Comparison of the microarray results obtained for shSLY and shSLX1shSLY round spermatids. List of the 222 genes showing greater than 1.5 fold-change in Sly-deficient spermatids (i.e. shSLY) relative to WT; 196 of them were corrected to some degree by addition of the shSLX transgene (in shSLX1shSLY). For 46 of these genes, the difference between shSLY and shSLX1shSLY was itself statistically significant. (PDF) [file pgen.1002900.s003.pdf]

Figure S3

Data normalisation and p value calculation were carried out in BeadStudio (Illumina), using the Illumina custom error model
This table shows all genes found to be 1.5fold regulated in shSLY relative to wild type, with an FDR corrected p value < 0.05
The rightmost columns indicate:
1) Whether the magnitude of the log ratio to WT was lower in shSLX1shSLY than in shSLY (i.e. correction of the de-regulation)
2) If so, whether the difference between shSLX1SLY and shSLY had an FDR corrected p value < 0.05

	shSLY		shSLX1shSLY		Annotation			Correction	
**SYMBOL**	**Log2_ratio**	**p(XY)**	**Log2_ratio**	**p(shSLY)**	**SEARCH_KEY**	**entrez_id**	**Chr.**	**Mag**	**Sig**
LOC271697	-0,609	0,000	-0,647	1,000	ILMN_196910	271697	1	N	-
Ufc1	-0,708	0,000	-0,812	0,890	ILMN_222108	66155	1	N	-
1110008P14Rik	1,017	0,000	0,520	0,002	ILMN_216202	73737	2	Y	Y
2210008N01Rik	0,861	0,000	0,771	1,000	ILMN_191351	76579	2	Y	N
Cebpb	0,689	0,002	0,190	0,138	ILMN_222901	12608	2	Y	N
Cst3	0,745	0,021	-0,007	0,077	ILMN_215837	13010	2	Y	N
LOC100045403	0,890	0,000	0,493	0,023	ILMN_221330	100045403	2	Y	Y
Phpt1	-0,951	0,000	-0,809	1,000	ILMN_212413	75454	2	Y	N
Slc39a12	-0,642	0,005	-0,068	0,071	ILMN_250798	277468	2	Y	N
Spinlw1	0,885	0,001	0,435	0,194	ILMN_212156	75526	2	Y	N
4933417G07Rik	-0,732	0,000	-0,626	1,000	ILMN_191795	71182	3	Y	N
Hsd3b4	-0,617	0,010	-0,187	0,201	ILMN_222998	15495	3	Y	N
Lxn	-0,771	0,005	-0,475	0,207	ILMN_218315	17035	3	Y	N
2410166I05Rik	-0,636	0,000	-0,787	1,000	ILMN_212736	76824	4	N	-
Camk2n1	-0,635	0,000	-0,491	0,913	ILMN_254160	66259	4	Y	N
LOC677215	-0,734	0,000	-0,847	1,000	ILMN_201550	665298	4	N	-
4930449I24Rik	0,787	0,009	0,540	0,734	ILMN_253759	67410	5	Y	N
6330405H19	0,651	0,048	0,883	1,000	ILMN_221175	100038441	5	N	-
LOC100044549	0,607	0,005	0,678	1,000	ILMN_224136	100044549	5	N	-
LOC639341	0,680	0,000	0,323	0,015	ILMN_198203	639341	5	Y	Y
Speer4a	0,777	0,017	0,295	0,311	ILMN_201519	75657	5	Y	N
Wsb2	0,737	0,000	0,400	0,427	ILMN_187961	59043	5	Y	N
Pex5	0,605	0,044	0,160	0,532	ILMN_209293	19305	6	Y	N
Egfbp2	0,772	0,029	0,221	0,218	ILMN_196757	13647	7	Y	N

Hnrpl	0,616	0,003	0,286	0,313	ILMN_212547	15388	7	Y	N
Klk1	1,016	0,000	0,867	1,000	ILMN_196747	16612	7	Y	N
Klk1b26	2,043	0,009	2,249	1,000	ILMN_196712	16618	7	N	-
Klk1b5	1,313	0,000	0,773	0,217	ILMN_196768	16622	7	Y	N
Klk1b9	1,081	0,036	1,046	0,878	ILMN_196709	13648	7	Y	N
LOC545952	0,987	0,002	0,421	0,418	ILMN_258759	100038914	7	Y	N
Tmem86a	1,017	0,000	0,777	0,233	ILMN_214221	67893	7	Y	N
Zfp36	0,801	0,027	0,150	0,196	ILMN_188430	22695	7	Y	N
1700096P03Rik	-0,765	0,000	-0,540	0,599	ILMN_188465	73557	8	Y	N
1810012K16Rik	-1,644	0,000	-1,130	0,063	ILMN_215805	69108	8	Y	N
LOC100047759	-0,975	0,001	-1,474	0,383	ILMN_201550	100042734	8	N	-
Mrpl34	0,635	0,008	0,077	0,043	ILMN_222071	94065	8	Y	Y
Rnf170	-0,879	0,000	-0,371	0,001	ILMN_187098	77733	8	Y	Y
9430081H08Rik	0,944	0,000	0,532	0,416	ILMN_191533	77430	9	Y	N
Btg4	1,083	0,000	0,824	0,466	ILMN_214539	56057	9	Y	N
Cd3d	0,600	0,000	0,248	0,000	ILMN_218570	12500	9	Y	Y
Cypt10	1,003	0,000	0,589	0,037	ILMN_236117	664726	9	Y	Y
Cypt9	0,926	0,019	0,360	0,304	ILMN_237115	664724	9	Y	N
Rplp1	-0,762	0,000	-0,855	1,000	ILMN_201550	56040	9	N	-
Usp3	0,715	0,000	0,493	1,000	ILMN_190457	235441	9	Y	N
4930528J18Rik	0,753	0,000	0,459	0,442	ILMN_187039	78195	10	Y	N
A630084D02Rik	0,639	0,000	0,353	0,090	ILMN_203888	70208	10	Y	N
1700092K14Rik	0,637	0,000	0,230	0,142	ILMN_219587	73536	11	Y	N
Rdm1	-0,686	0,000	-0,753	1,000	ILMN_215276	66599	11	N	-
Esrrb	-0,597	0,011	-0,469	1,000	ILMN_213635	26380	12	Y	N
Agtr1a	-1,455	0,000	-0,829	0,018	ILMN_222907	11607	13	Y	Y
Hist1h3a	0,689	0,000	0,486	0,977	ILMN_246403	360198	13	Y	N
Hist1h3d	0,640	0,004	0,589	1,000	ILMN_187783	319149	13	Y	N
Hist1h3e	0,663	0,000	0,615	1,000	ILMN_240054	319151	13	Y	N
Hist1h3h	0,587	0,000	0,820	0,946	ILMN_249613	319152	13	N	-
1500001L15Rik	-0,725	0,000	-0,590	1,000	ILMN_221574	68966	14	Y	N
1700080P15Rik	-0,872	0,003	-0,400	0,452	ILMN_202196	18530	14	Y	N
E330034G19Rik	0,780	0,000	0,739	0,979	ILMN_196636	105418	14	Y	N
EG545047	0,941	0,039	1,051	1,000	ILMN_225380	545047	14	N	-
EG546250	0,967	0,002	0,811	1,000	ILMN_201457	546250	14	Y	N
EG638262	0,677	0,000	0,404	0,257	ILMN_201460	638262	14	Y	N

Fndc3	-0,998	0,000	-0,774	0,688	ILMN_188804	319448	14	Y	N
LOC100040863	0,756	0,000	0,221	0,483	ILMN_199680	100040863	14	Y	N
LOC544988	0,649	0,002	0,161	0,196	ILMN_230723	100042149	14	Y	N
2010001J22Rik	0,764	0,004	0,167	0,227	ILMN_218106	70113	15	Y	N
Ext1	-0,707	0,000	-0,605	1,000	ILMN_221180	14042	15	Y	N
Gpr172b	-0,685	0,000	-0,448	0,501	ILMN_210952	52710	15	Y	N
Prr13	-0,917	0,000	-0,851	1,000	ILMN_215277	66151	15	Y	N
Chaf1b	-0,721	0,000	-0,779	1,000	ILMN_215876	110749	16	N	-
Efcab1	0,636	0,012	0,691	1,000	ILMN_216541	66793	16	N	-
ENSMUSG00000044227	0,949	0,047	0,382	0,474	ILMN_206641	640627	16	Y	N
Tbx1	0,773	0,010	0,383	0,690	ILMN_222134	21380	16	Y	N
Alkbh7	-0,659	0,000	-0,470	0,831	ILMN_218238	66400	17	Y	N
Def6	0,674	0,017	0,117	0,209	ILMN_209446	23853	17	Y	N
Rpp21	-0,873	0,000	-0,745	0,847	ILMN_220751	67676	17	Y	N
Salf-pending	-0,957	0,000	-0,998	1,000	ILMN_201586	77057	17	N	-
2400010D15Rik	-0,718	0,000	-0,589	1,000	ILMN_211381	76482	18	Y	N
4933426E01Rik	-0,619	0,037	-0,799	1,000	ILMN_202859	13510	18	N	-
Sra1	-0,651	0,001	-0,509	1,000	ILMN_208746	24068	18	Y	N
Btrc	-0,821	0,003	-0,423	0,686	ILMN_193084	12234	19	Y	N
1110059M19Rik	0,918	0,000	0,079	0,004	ILMN_220773	68800	X	Y	Y
1600025M17Rik	1,513	0,000	0,698	0,004	ILMN_221630	72030	X	Y	Y
1700006H02Rik	1,070	0,000	1,122	1,000	ILMN_196351	69361	X	N	-
1700008I05Rik	1,156	0,000	0,848	0,281	ILMN_216883	71841	X	Y	N
1700010D01Rik	1,186	0,000	1,124	1,000	ILMN_211201	76386	X	Y	N
1700013H16Rik	0,783	0,036	0,736	1,000	ILMN_217112	75514	X	Y	N
1700020N15Rik	0,856	0,006	0,199	0,065	ILMN_220159	75509	X	Y	N
1700025D03Rik	1,214	0,000	0,756	0,143	ILMN_201762	67944	X	Y	N
1700031F05Rik	0,742	0,000	0,595	1,000	ILMN_196511	73300	X	Y	N
1700042B14Rik	0,958	0,000	0,694	0,008	ILMN_216906	73347	X	Y	Y
1700045I19Rik	0,726	0,000	0,399	0,198	ILMN_209606	74264	X	Y	N
1700054O13Rik	0,603	0,000	0,464	1,000	ILMN_221831	67334	X	Y	N
1700072E05Rik	0,837	0,000	1,094	0,574	ILMN_209155	73495	X	N	-
1700080O16Rik	1,034	0,000	0,845	0,654	ILMN_209046	74279	X	Y	N
1700084M14Rik	1,006	0,000	0,450	0,024	ILMN_184803	73487	X	Y	Y
1700085A12Rik	1,517	0,017	1,183	1,000	ILMN_193185	78631	X	Y	N
1700123J19Rik	0,610	0,016	0,448	1,000	ILMN_210758	73614	X	Y	N

1700129I15Rik	0,729	0,003	1,013	0,262	ILMN_185761	78478	X	N	-
3110007F17Rik	1,358	0,000	1,074	0,877	ILMN_188622	73061	X	Y	N
4930428D18Rik	1,339	0,000	0,765	0,005	ILMN_225536	619294	X	Y	Y
4930428E23Rik	0,960	0,000	0,701	0,427	ILMN_244828	434800	X	Y	N
4930430D24Rik	1,089	0,000	0,319	0,094	ILMN_256260	194735	X	Y	N
4930432H15Rik	1,006	0,000	0,772	0,922	ILMN_219215	73866	X	Y	N
4930447F04Rik	0,744	0,000	0,610	1,000	ILMN_196508	74862	X	Y	N
4930468A15Rik	1,237	0,000	1,121	1,000	ILMN_218851	74987	X	Y	N
4930503H13Rik	0,810	0,017	0,305	0,376	ILMN_187318	74941	X	Y	N
4930519F16Rik	1,215	0,000	0,534	0,019	ILMN_194173	75106	X	Y	Y
4930524E20Rik	1,102	0,000	0,406	0,000	ILMN_242965	75097	X	Y	Y
4930524N10Rik	0,862	0,000	0,631	0,262	ILMN_196516	75071	X	Y	N
4930557A04Rik	1,033	0,000	0,376	0,001	ILMN_217036	385317	X	Y	Y
4933400A11Rik	0,917	0,002	0,922	1,000	ILMN_221738	66747	X	N	-
4933403O08Rik	0,663	0,019	0,365	0,884	ILMN_214452	71030	X	Y	N
4933416I08Rik	0,760	0,001	0,147	0,124	ILMN_219534	71159	X	Y	N
Abcd1	0,628	0,000	0,295	0,383	ILMN_209991	11666	X	Y	N
Actrt1	1,639	0,000	1,124	0,279	ILMN_211644	73360	X	Y	N
Akap14	1,031	0,000	0,673	0,006	ILMN_241953	434756	X	Y	Y
Arhgef6	1,122	0,006	0,721	0,748	ILMN_202183	73341	X	Y	N
Arl13a	1,106	0,002	0,946	1,000	ILMN_223123	74448	X	Y	N
Asb12	1,192	0,000	0,352	0,037	ILMN_219860	70392	X	Y	Y
Asb9	0,741	0,004	0,135	0,074	ILMN_217406	69299	X	Y	N
Cdkl5	0,731	0,000	0,702	1,000	ILMN_213972	382253	X	Y	N
Cnbp2	1,164	0,000	0,561	0,000	ILMN_258248	75064	X	Y	Y
Cpxcr1	1,456	0,005	1,284	1,000	ILMN_232377	382239	X	Y	N
Ctag2	1,514	0,000	1,421	1,000	ILMN_222168	70062	X	Y	N
Cypt2	1,424	0,000	0,757	0,000	ILMN_223630	245566	X	Y	Y
Cypt3	0,941	0,000	0,843	0,946	ILMN_196351	69361	X	Y	N
Cypt6	1,010	0,000	0,897	1,000	ILMN_220801	66742	X	Y	N
Dmrtc1a	0,810	0,000	0,581	0,480	ILMN_202428	70887	X	Y	N
Dmrtc1c	0,799	0,000	0,623	0,801	ILMN_217002	71083	X	Y	N
EG210457	1,517	0,000	1,213	0,427	ILMN_198549	210457	X	Y	N
EG237009	1,818	0,047	1,760	1,000	ILMN_241004	237009	X	Y	N
EG238829	0,969	0,006	1,042	1,000	ILMN_198726	238829	X	N	-
EG382265	1,317	0,000	0,811	0,072	ILMN_200946	382265	X	Y	N

EG546347	0,864	0,000	0,716	1,000	ILMN_232085	546347	X	Y	N	
EG627311	1,072	0,000	0,194	0,025	ILMN_198707	627311	X	Y	Y	
EG668360	1,152	0,000	0,949	1,000	ILMN_200842	668360	X	Y	N	
F8a	0,974	0,000	0,520	0,307	ILMN_194941	14070	X	Y	N	
Fmr1nb	0,619	0,000	0,310	0,092	ILMN_190519	207854	X	Y	N	
Gm1141	0,630	0,028	0,844	1,000	ILMN_200818	382221	X	N	-	
Gm362	1,144	0,000	0,664	0,353	ILMN_198555	236774	X	Y	N	
Gm47	0,602	0,000	-0,018	0,143	ILMN_198704	195726	X	Y	N	
Gm732	1,086	0,000	0,869	0,855	ILMN_260916	213450	X	Y	N	
Gm773	0,917	0,015	0,824	1,000	ILMN_224498	331416	X	Y	N	
Gspt2	0,677	0,000	0,591	1,000	ILMN_185166	14853	X	Y	N	
Klhl15	0,772	0,000	0,568	0,064	ILMN_188378	236904	X	Y	N	
LOC100040020	1,155	0,000	0,674	0,013	ILMN_223941	100040020	X	Y	Y	
LOC100040899	0,837	0,000	0,698	1,000	ILMN_254706	100040899	X	Y	N	
LOC211702	1,166	0,000	0,799	0,682	ILMN_198612	211702	X	Y	N	
LOC236749	1,132	0,000	0,903	1,000	ILMN_236473	236749	X	Y	N	
LOC238829	1,145	0,000	1,045	1,000	ILMN_255176	238829	X	Y	N	
LOC245600	0,926	0,000	0,683	0,875	ILMN_198764	245600	X	Y	N	
LOC245693	0,873	0,000	0,547	0,186	ILMN_198715	245693	X	Y	N	
LOC270621	0,697	0,000	0,366	0,286	ILMN_198743	667934	X	Y	N	
LOC331528	0,586	0,000	0,479	1,000	ILMN_198669	331528	X	Y	N	
LOC333588	1,419	0,000	0,454	0,070	ILMN_198688	333588	X	Y	N	
LOC382213	0,990	0,000	-0,826	0,000	ILMN_200813		X	Y	Y	
LOC382232	1,017	0,000	0,685	0,686	ILMN_200841	100042465	X	Y	N	
LOC385360	0,776	0,004	0,706	1,000	ILMN_200820	385360	X	Y	N	
LOC631002	0,814	0,000	0,554	0,659	ILMN_252375	631002	X	Y	N	
LOC631784	1,136	0,024	1,122	1,000	ILMN_257790	631784	X	Y	N	
LOC634955	1,230	0,000	0,695	0,013	ILMN_200784	628871	X	Y	Y	
LOC666096	1,165	0,000	-0,373	0,000	ILMN_224045	666096	X	Y	Y	
Maged1	0,782	0,000	0,783	1,000	ILMN_215570	94275	X	N	-	
Morf4l2	0,819	0,000	0,514	0,366	ILMN_209831	56397	X	Y	N	
Ott	0,854	0,000	0,946	1,000	ILMN_221772	18422	X	N	-	
OTTMUSG00000016790	0,680	0,003	0,163	0,140	ILMN_236251	547160	X	Y	N	
OTTMUSG00000017677	1,071	0,000	0,836	0,136	ILMN_237635	574404	X	Y	N	
OTTMUSG00000017827	1,413	0,000	0,696	0,112	ILMN_231407	628053	X	Y	N	
OTTMUSG00000019001	0,719	0,006	0,551	1,000	ILMN_232603	434864	X	Y	N	

Pdk3	0,695	0,000	0,487	0,770	ILMN_216915	236900	X	Y	N
Pja1	0,713	0,000	0,625	0,669	ILMN_211345	18744	X	Y	N
Ppef1	0,739	0,000	0,089	0,037	ILMN_198711	237178	X	Y	Y
Prdx4	0,703	0,001	0,736	1,000	ILMN_201946	53381	X	N	-
Rhox11	1,624	0,000	0,899	0,008	ILMN_223196	194738	X	Y	Y
Rhox3f	0,854	0,002	0,255	0,056	ILMN_239358	621852	X	Y	N
Ribc1	0,853	0,000	0,744	0,922	ILMN_217863	66611	X	Y	N
Rp23-438h3.2	1,349	0,010	1,214	1,000	ILMN_212722	69357	X	Y	N
Satl1	0,981	0,000	0,568	0,009	ILMN_219886	73809	X	Y	Y
Sh3kbp1	0,983	0,000	0,617	0,345	ILMN_219487	58194	X	Y	N
Slc10a3	0,850	0,000	0,593	0,648	ILMN_209098	214601	X	Y	N
Spaca5	1,135	0,032	0,097	0,080	ILMN_198542	278203	X	Y	N
Ssxb2	0,655	0,042	0,469	0,998	ILMN_233470	387132	X	Y	N
Ssxb5	0,654	0,000	0,364	0,484	ILMN_228620	387586	X	Y	N
Sytl5	0,784	0,000	0,523	0,114	ILMN_222257	236643	X	Y	N
Tgif2lx	1,596	0,000	1,457	1,000	ILMN_221737	245583	X	Y	N
Tmsb4x	1,013	0,000	0,685	0,111	ILMN_196070	19241	X	Y	N
Ube2dnl	1,660	0,000	0,691	0,000	ILMN_221710	237009	X	Y	Y
Ubl4	0,730	0,020	0,054	0,043	ILMN_189969	27643	X	Y	Y
Wdr13	0,848	0,000	0,745	1,000	ILMN_186433	73447	X	Y	N
Xk	0,604	0,011	0,235	0,452	ILMN_188482	22439	X	Y	N
Zcchc13	1,323	0,000	0,530	0,003	ILMN_196673	75064	X	Y	Y
Zic3	0,702	0,000	0,629	1,000	ILMN_186994	22773	X	Y	N
Zmym3	0,868	0,007	0,676	0,937	ILMN_185339	56364	X	Y	N
LOC100039516	0,834	0,000	0,622	0,156	ILMN_201035	100039516	Y	Y	N
LOC100039829	0,789	0,000	0,574	0,081	ILMN_201036	100039829	Y	Y	N
LOC100040384	0,837	0,000	0,395	0,000	ILMN_201013	100040384	Y	Y	Y
LOC100041256	1,012	0,000	0,510	0,001	ILMN_201014	100041256	Y	Y	Y
LOC100041608	1,280	0,010	0,368	0,119	ILMN_199487	100041608	Y	Y	N
LOC100041934	0,701	0,000	0,438	0,019	ILMN_201008	100041934	Y	Y	Y
LOC100041980	1,204	0,000	0,540	0,000	ILMN_201020	100041980	Y	Y	Y
LOC100042145	0,980	0,021	0,255	0,206	ILMN_201027	100042145	Y	Y	N
LOC100042290	1,324	0,000	0,924	0,000	ILMN_201017	100042290	Y	Y	Y
LOC100042351	0,932	0,000	0,452	0,000	ILMN_201019	100042351	Y	Y	Y
LOC100042481	1,244	0,000	0,765	0,004	ILMN_201553	100042481	Y	Y	Y
LOC100042550	1,060	0,000	0,552	0,000	ILMN_201012	100042550	Y	Y	Y

LOC380994	0,602	0,028	0,445	1,000	ILMN_232232	380994	Y	Y	N
LOC382133	1,180	0,000	0,670	0,000	ILMN_250371	382133	Y	Y	Y
LOC382297	0,673	0,000	0,513	0,307	ILMN_201007		Y	Y	N
LOC385542	1,323	0,000	0,612	0,037	ILMN_201010		Y	Y	Y
LOC385543	1,252	0,000	0,913	0,913	ILMN_201011		Y	Y	N
LOC385583	1,261	0,000	0,796	0,001	ILMN_201031		Y	Y	Y
LOC434960	0,840	0,001	0,071	0,010	ILMN_240151	434960	Y	Y	Y
LOC665746	1,287	0,000	0,513	0,016	ILMN_201018	665746	Y	Y	Y
LOC665918	1,183	0,000	0,482	0,000	ILMN_201016	665918	Y	Y	Y
MGC107098	1,231	0,001	1,185	1,000	ILMN_255921	434935	Y	Y	N
Rbm31y	0,862	0,002	0,750	1,000	ILMN_188939	74484	Y	Y	N
Ssty1	1,294	0,000	0,795	0,002	ILMN_201553	20611	Y	Y	Y
Ssty2	1,036	0,000	0,429	0,186	ILMN_223346	70009	Y	Y	N
4732458O05Rik	-0,588	0,000	-0,786	1,000	ILMN_203137			N	-
E030024C07Rik	0,845	0,022	0,783	1,000	ILMN_205793			Y	N
LOC100045423	0,989	0,000	0,492	0,052	ILMN_213375	100045423		Y	N
LOC672441	1,418	0,000	0,881	0,542	ILMN_198839	672441		Y	N
LOC673063	1,215	0,000	0,758	0,597	ILMN_196700	673063		Y	N
							222	196	46
