# Supplementary material for: A Genetic Basis for a Postmeiotic X Versus Y Chromosome Intragenomic Conflict in the Mouse
Source: PLoS Genet. 2012 Sep 13;8(9):e1002900. doi: 10.1371/journal.pgen.1002900 (PMC3441658; doi:10.1371/journal.pgen.1002900)
Supplement: Figure S5 — Comparison of the microarray results obtained for shSLX1 and shSLX1shSLY round spermatids. List of the 115 genes showing greater than 1.5 fold-change in Slx/Slxl1-deficient spermatids (i.e. shSLX1) relative to WT; 111 of them were corrected to some degree by addition of the shSLY transgene (in shSLX1shSLY). For 91 of these genes, the difference between shSLX1 and shSLX1shSLY was itself statistically significant. (PDF) [file pgen.1002900.s005.pdf]

Figure S5

This table shows all genes found to be 1.5fold regulated in shSLX1 relative to wild type, with an FDR corrected p value < 0.05
The rightmost columns indicate:

1) Whether the magnitude of the log ratio to WT was lower in shSLX1shSLY than in shSLX1 (i.e. correction of the de-regulation)
2) If so, whether the difference between shSLX1SLY and shSLX1 had an FDR corrected p value < 0.05

	shSLX1		shSLX1shSLY		Annotation			Correction	
SYMBOL	Log2_ratio	p(XY)	Log2_ratio	p(shSLX1)	SEARCH_KEY	entrez_id	Chr.	Mag	Sig
Aox4	1,227	0,000	0,043	0,000	ILMN_222582	71872	1	Y	Y
Atp6v1h	0,860	0,000	0,264	0,000	ILMN_221485	108664	1	Y	Y
Fam178b	-0,656	0,000	-0,803	0,886	ILMN_217514	381337	1	N	-
Fhl2	0,618	0,001	0,055	0,002	ILMN_186128	14200	1	Y	Y
LOC100043347	0,671	0,000	-0,161	0,000	ILMN_215832	100043347	1	Y	Y
Slamf9	1,104	0,000	-0,148	0,000	ILMN_215754	98365	1	Y	Y
1110008P14Rik	0,648	0,005	0,520	1,000	ILMN_216202	73737	2	Y	N
1700058C13Rik	0,648	0,000	-0,005	0,001	ILMN_222471	73388	2	Y	Y
Agpat2	0,632	0,001	-0,160	0,000	ILMN_217418	67512	2	Y	Y
Angptl2	0,632	0,017	-0,078	0,024	ILMN_202070	26360	2	Y	Y
Bmp7	1,251	0,000	-0,066	0,000	ILMN_202602	12162	2	Y	Y
Col20a1	0,786	0,000	0,093	0,000	ILMN_209312	73368	2	Y	Y
Ctdspl2	0,702	0,000	0,021	0,000	ILMN_197409	329506	2	Y	Y
Defb21	1,355	0,000	-0,462	0,000	ILMN_217555	403172	2	Y	Y
Lcn2	0,626	0,000	0,104	0,002	ILMN_219674	16819	2	Y	Y
Nudt5	1,473	0,000	0,094	0,000	ILMN_213908	53893	2	Y	Y
Rapgef4	0,723	0,000	0,063	0,001	ILMN_213156	56508	2	Y	Y
Chi3l3	1,692	0,000	0,034	0,000	ILMN_226085	12655	3	Y	Y
Chi3l4	1,315	0,000	-0,152	0,000	ILMN_196569	104183	3	Y	Y
Dennd4b	-0,715	0,044	-0,396	0,030	ILMN_184356	229541	3	Y	Y
Larp1b	0,959	0,000	0,093	0,000	ILMN_216009	214048	3	Y	Y
Lrriq3	0,640	0,049	0,391	0,365	ILMN_202332	74435	3	Y	N
Tmod4	1,857	0,000	-0,370	0,000	ILMN_186822	50874	3	Y	Y
Ambp	0,906	0,000	0,116	0,008	ILMN_218973	11699	4	Y	Y

BC055111	0,715	0,048	0,324	0,787	ILMN_215845	242602	4	Y	N
Cela2a	0,612	0,000	-0,082	0,000	ILMN_216734	13706	4	Y	Y
Guca2a	1,211	0,000	-0,320	0,000	ILMN_213266	14915	4	Y	Y
Ppap2b	1,079	0,000	-0,056	0,000	ILMN_212849	67916	4	Y	Y
Abcb1b	0,606	0,000	-0,021	0,000	ILMN_212008	18669	5	Y	Y
Gnrhr	0,696	0,000	0,156	0,001	ILMN_185817	14715	5	Y	Y
Hgfac	0,800	0,000	0,093	0,000	ILMN_218356	54426	5	Y	Y
Myl2	1,514	0,000	-0,021	0,000	ILMN_224025	17906	5	Y	Y
4933413G19Rik	0,851	0,006	0,090	0,052	ILMN_189726	71149	6	Y	N
Hipk2	0,600	0,001	-0,001	0,001	ILMN_201896	15258	6	Y	Y
St3gal5	0,602	0,002	-0,140	0,000	ILMN_219818	20454	6	Y	Y
Actn4	0,915	0,001	-0,394	0,000	ILMN_212299	60595	7	Y	Y
Cyp2a12	0,885	0,002	-0,103	0,000	ILMN_214236	13085	7	Y	Y
Klk1	1,500	0,000	0,867	0,101	ILMN_196747	16612	7	Y	N
Klk1b26	1,752	0,000	2,249	0,377	ILMN_196712	16618	7	N	-
Klk1b4	0,815	0,000	0,077	0,000	ILMN_199361	18048	7	Y	Y
Klk1b5	1,590	0,000	0,773	0,016	ILMN_196768	16622	7	Y	Y
Klk1b9	1,020	0,005	1,046	0,544	ILMN_196709	13648	7	N	-
Lilra6	0,762	0,000	-0,100	0,000	ILMN_201549	18726	7	Y	Y
Mettl9	0,771	0,001	0,335	0,476	ILMN_219776	59052	7	Y	N
Pira2	1,197	0,000	0,019	0,000	ILMN_189324	18725	7	Y	Y
Shank2	0,810	0,000	0,212	0,056	ILMN_211643	210274	7	Y	N
Ccl17	0,655	0,004	0,021	0,003	ILMN_220052	20295	8	Y	Y
Col4a2	0,994	0,000	-0,006	0,000	ILMN_216595	12827	8	Y	Y
F10	0,687	0,000	0,036	0,033	ILMN_217890	14058	8	Y	Y
Fam70b	0,779	0,000	-0,092	0,000	ILMN_198849	272465	8	Y	Y
Jund	-0,766	0,000	0,404	0,000	ILMN_217403	16478	8	Y	Y
LOC100045280	0,817	0,020	0,049	0,034	ILMN_203136	100045280	8	Y	Y
Plcg2	0,625	0,020	-0,074	0,000	ILMN_210061	234779	8	Y	Y
Rnf170	0,810	0,000	-0,371	0,000	ILMN_187098	77733	8	Y	Y
Tnfsf13b	0,762	0,000	0,141	0,000	ILMN_184661	24099	8	Y	Y
1700019J19Rik	1,014	0,000	-0,183	0,000	ILMN_221740	76412	9	Y	Y
Cmc1	0,723	0,003	-0,201	0,000	ILMN_192959	67899	9	Y	Y
Fxyd2	0,590	0,001	0,089	0,048	ILMN_224097	11936	9	Y	Y
Myl3	0,880	0,003	0,134	0,010	ILMN_218112	17897	9	Y	Y
Ntm	0,632	0,004	-0,215	0,001	ILMN_184137	235106	9	Y	Y

Pstpip1	0,710	0,000	-0,273	0,000	ILMN_215958	19200	9	Y	Y
Usp3	0,836	0,000	0,493	0,733	ILMN_190457	235441	9	Y	N
Gm1552	0,687	0,000	-0,071	0,000	ILMN_199038	380640	10	Y	Y
LOC100044873	0,829	0,000	0,045	0,001	ILMN_215989	100044873	10	Y	Y
Prep	0,653	0,012	0,059	0,014	ILMN_202422	19072	10	Y	Y
Ube2g2	0,720	0,000	-0,092	0,000	ILMN_188977	22213	10	Y	Y
Aatf	0,654	0,001	-0,218	0,000	ILMN_212076	56321	11	Y	Y
Arrb2	0,924	0,000	-0,066	0,000	ILMN_218837	216869	11	Y	Y
B3gntl1	0,698	0,000	-0,257	0,000	ILMN_208620	210004	11	Y	Y
Ccdc57	0,772	0,000	0,076	0,000	ILMN_223765	71276	11	Y	Y
Ccl5	0,682	0,018	0,046	0,017	ILMN_212715	20304	11	Y	Y
Fbll1	0,690	0,003	0,346	0,456	ILMN_199174	237730	11	Y	N
LOC100044934	0,761	0,000	-0,082	0,000	ILMN_221227	100044934	11	Y	Y
LOC100045019	0,649	0,000	-0,195	0,000	ILMN_195453	100045019	11	Y	Y
Tmem11	0,612	0,000	-0,261	0,000	ILMN_214265	216821	11	Y	Y
Zmat5	1,138	0,000	-0,311	0,000	ILMN_218036	67178	11	Y	Y
4933437F05Rik	0,875	0,000	0,536	0,745	ILMN_220294	71275	12	Y	N
Serpina1d	0,834	0,000	-0,118	0,000	ILMN_222172	20703	12	Y	Y
0610007P08Rik	0,716	0,000	-0,017	0,000	ILMN_201703	76251	13	Y	Y
LOC100046232	1,215	0,000	-0,147	0,000	ILMN_209434	100046232	13	Y	Y
Nqo2	0,730	0,002	0,130	0,116	ILMN_208782	18105	13	Y	N
Pom121l2	-0,608	0,025	-0,572	1,000	ILMN_197161	195236	13	Y	N
Pou6f2	1,346	0,000	0,290	0,000	ILMN_204071	218030	13	Y	Y
Tgfbi	1,084	0,000	-0,110	0,000	ILMN_192154	21810	13	Y	Y
Fhit	1,189	0,000	0,275	0,000	ILMN_222375	14198	14	Y	Y
Fndc3a	1,104	0,000	-0,774	0,000	ILMN_188804	319448	14	Y	Y
Gulo	1,228	0,002	-0,025	0,001	ILMN_212949	268756	14	Y	Y
Fam186a	-0,594	0,001	-0,625	1,000	ILMN_197658	380973	15	N	-
Gsdmd	0,908	0,000	-0,036	0,000	ILMN_222389	69146	15	Y	Y
Hoxc9	0,940	0,000	0,446	0,046	ILMN_223992	15427	15	Y	Y
Nfe2	0,667	0,000	-0,077	0,000	ILMN_220449	18022	15	Y	Y
Efcab1	0,732	0,000	0,691	1,000	ILMN_216541	66793	16	Y	N
Stfa1	1,870	0,000	0,066	0,000	ILMN_245739	20861	16	Y	Y
4930583I09Rik	1,018	0,000	0,029	0,000	ILMN_219612	78057	17	Y	Y
Strn	0,938	0,000	0,037	0,000	ILMN_184393	268980	17	Y	Y
Tff2	0,601	0,020	-0,094	0,004	ILMN_216698	21785	17	Y	Y

Thbs2	1,011	0,000	-0,187	0,000	ILMN_213253	21826	17	Y	Y	
Arhgap26	0,941	0,000	-0,001	0,000	ILMN_201922	71302	18	Y	Y	
St8sia5	0,768	0,000	0,053	0,000	ILMN_218246	225742	18	Y	Y	
Acsl5	0,945	0,029	-0,161	0,029	ILMN_189230	433256	19	Y	Y	
AK076554	0,762	0,000	-0,195	0,000	ILMN_206634	208449	19	Y	Y	
Gpr120	1,099	0,000	-0,101	0,000	ILMN_222251	107221	19	Y	Y	
Pold4	0,793	0,000	-0,319	0,000	ILMN_233673	69745	19	Y	Y	
Rps6ka4	-0,718	0,001	-0,583	1,000	ILMN_221814	56613	19	Y	N	
Dusp21	-0,606	0,034	0,296	0,001	ILMN_222304	73547	X	Y	Y	
Gm14525	-0,800	0,048	0,285	0,038	ILMN_192739	100039120	X	Y	Y	= Slx/Slxl1
Gm14594	-1,120	0,000	-0,373	0,338	ILMN_224045	666096	X	Y	N	= Slx/Slxl1
Gm5168	-1,001	0,000	-0,477	1,000	ILMN_232052	382275	X	Y	N	= Slx/Slxl1
Gm5935	-0,861	0,003	-0,618	1,000	ILMN_235895	546282	X	Y	N	= Slx/Slxl1
LOC382213	-1,212	0,000	-0,826	1,000	ILMN_200813		X	Y	N	= Slx/Slxl1
mt-Nd5	0,969	0,018	0,750	1,000	ILMN_194230	17721	MT	Y	N	
AK039094	0,732	0,000	-0,233	0,000	ILMN_204680			Y	Y	
Cgef2-pending	0,642	0,000	0,246	0,078	ILMN_201828			Y	N	
LOC236371	0,715	0,000	0,301	0,012	ILMN_199291			Y	Y	
scl0001883.1_112	0,670	0,000	0,054	0,000	ILMN_193472			Y	Y	
							115	111	91	
