# Supplementary material for: A Genetic Basis for a Postmeiotic X Versus Y Chromosome Intragenomic Conflict in the Mouse
Source: PLoS Genet. 2012 Sep 13;8(9):e1002900. doi: 10.1371/journal.pgen.1002900 (PMC3441658; doi:10.1371/journal.pgen.1002900)
Supplement: Table S1 — List of the primers used in the study. (PDF) [file pgen.1002900.s008.pdf]

Table S1. List of the primers used in the study

		**Primers used to produce tagged constructs**	
**Construct**	**Primer name**	**Sequence**	
pCMV-Dkkl1-myc	pCMV-Dkkl1-Eco-myc-F	AAAAGAATTCGCAATGTGTCGACTGAGGGTC	
	pCMV-Dkkl1-Msc-myc-Xho-R	AAAATGGCCATCATTCATTCAAGTCCTCTTCAGAAATGAGCTTTTGCTCCATCTCGAGCAGCTGTTGGGATGGCCTGAG	
pCMV-Slx-myc	pCMV-Not-Slx-F	AAA AGCGGCCGCATGTCTATTAAGAAACTGTGGGT	
	pCMV-MscI-Myc Slx-R	AAAATGGCCATCATTCATTCAAGTCCTCTTCAGAAATGAGCTTTTGCTCCATTAATGTCTCTTCACCATCTACAT	
pCMV-Flag-Sly1	pCMV-Eco-SlyF	ATGGACGAATTCATGGCTCTTAAGAAATTGAAGGT	
	pCMV-NotI-Sly-R	CGAGTGCGGCCGCTTAGTTCTTG	
pCMV-Flag-Sly2	pCMV-Eco-SlyF	ATGGACGAATTCATGGCTCTTAAGAAATTGAAGGT	
	pCMV-NotI-Sly-R	CGAGTGCGGCCGCTTAGTTCTTG	
pCMV-Flag-Slx	pCMV-Eco-SlxF	ATTTGAATTCATGTCTATTAAGAAACTGTGGG	
	pCMV-NotI-Slx-R	AATTGCGGCCGCTCATAATGTCTCTTCACCATCTACA	
pCMV-Flag-Slxl1	pCMV-Eco-Slxl1F	ATTTGAATTCATGGCTCTTAAGAAACTGTGGG	
	pCMV-NotI-Slxl1-R	AATTGCGGCCGCTCATTTTCTCAATTCACCATCTACA	
		**Real time PCR primers**	
**Gene name**	**Primer name**	**Sequence**	**Reference**
Acrv1 (aka *SP-10*)			[19]
	Acrv1-F	TGAGTACACCACTTCCAAGCA	
	Acrv1-R	AAGCACATGTGTGGCAATTT	
Slx			[26]
	Slx-F	TTCAGATGAAGAAGAAGAGCAGG	
	Slx-R	TCCATATCAAACTTCTGCTCACAC	
Slxl1			[26]
	Slxl1-F	TTGGAGGACGCTCATTCTG	
	Slxl1-R	ACGACTTGTTGTTGATCATCTCC	
Slx/Slxl1 (Slx all)			[28]

	Slx-all-F	TTTCTCAGAGGAATGGCAGCG	
	Slx-all-R	TCATCATGAGTTACTGGATTCTGT	
Actrt1			[19]
	Actrt1-F	CTCAAAAATGGTCTGCAACAGC	
	Actrt1-R	TCTTGATAGGGGTTCCCTCAAA	
1700008I05Rik			[19]
	1700008I05Rik-F	AAAGCCAATTCGTGGAGACAAT	
	1700008I05Rik-R	TGGGAGAGATGCAGAATATCCA	
Ssty1			[26]
	Ssty1-F	AGAAGGATCCAGCTCTCTATGCT	
	Ssty1-R	CCAGTTACCAATCAACACATCAC	
Zfy2			[19]
	Zfy2-F	CTTAATTCCAGACATTTTAACTTCCA	
	Zfy2-R	ATCACTTGTTCAAAATGTCCTACATT	
Sly (*Sly1+2*)			[19]
	Global Sly F	CATTTATAAGACGCTTCACATAAAG	
	Global Sly R1	TCCTCCATGATGGCTCTTTC	
	Global Sly R2	ATTCTCCATGATGGCTCTTTC	
Sly			[26]
	Sly-F	CTGGAGATGACATTTATAAGACGC	
	Sly-R	TCCTCCATGATGGCTCTTTC	
